# Pain in recessive dystrophic epidermolysis bullosa (RDEB): findings of the Prospective Epidermolysis Bullosa Longitudinal Evaluation Study (PEBLES)

**DOI:** 10.1186/s13023-024-03349-w

**Published:** 2024-10-11

**Authors:** Eunice Jeffs, Elizabeth I. Pillay, Lesedi Ledwaba-Chapman, Alessandra Bisquera, Susan J. Robertson, John A. McGrath, Yanzhong Wang, Anna E. Martinez, Jemima E. Mellerio

**Affiliations:** 1 St. John’s Institute of Dermatology , Guy’s and St Thomas’ NHS Foundation Trust, London, UK; 2 Department of Population Health Sciences, King’s College London, London, UK; 3 Departments of Dermatology, The Royal Children’s Hospital, The Royal Melbourne Hospital and Murdoch Children’s Research Institute, Melbourne, Australia; 4 Genetic Skin Disease Group, King’s College London, London, UK; 5 Department of Dermatology, Great Ormond Street Hospital for Children NHS Foundation Trust, London, UK

**Keywords:** Epidermolysis bullosa, Pain, Natural history, Quality of life, Disease severity

## Abstract

**Background:**

Pain is common in the genetic skin fragility disorder epidermolysis bullosa (EB), from skin and mucosal injury and inflammation as well as extra-mucocutaneous sites. Individuals living with EB have identified pain as a priority for better treatments.

**Objectives:**

The Prospective EB Longitudinal Evaluation Study (PEBLES) is a prospective register study exploring the natural history of RDEB across all ages from birth to death. Here, we investigated the characteristics and treatment of pain in different RDEB subtypes.

**Methods:**

Information was collected from individuals with different RDEB subtypes over an 8-year period. Data included visual analogue scale (VAS) ratings of background and procedural pain, its location, intensity and impact on sleep, as well as pain medication. Disease severity scores and quality of life measures were correlated to pain scores.

**Results:**

Sixty-one participants (13 children, 48 adults) completed a total of 361 reviews. Pain was common, experienced by 93% of participants at index review, with 80% suffering both background and procedural pain. Across all RDEB patients, the median VAS for background pain was 40 (out of 100) [interquartile range 20,60] and for those having regular dressing changes, median procedural pain was 52 [40,80]. Severe (RDEB-S) and pruriginosa (RDEB-Pru) groups had the greatest increase in procedural compared to background pain of 20 and 22 VAS points, respectively. Correlations between disease severity and quality of life impairment were observed across most groups, particularly RDEB-S. Over half of those studied experienced pain frequently or constantly, and in one third pain disturbed sleep at least 4 nights per week. Skin was the commonest source of pain in all subtypes except inversa RDEB where the mouth was the main site. Despite frequent and severe pain, one third of participants used no medication for pain and, in those that did, pain levels remained high suggesting ineffectiveness of current pain management approaches and a significant unmet need in RDEB.

**Conclusion:**

The frequency, severity, and impact of pain in all RDEB patients is significant, particularly in RDEB-S and RDEB-Pru. Our findings highlight that current RDEB pain management is poorly effective and that further research is needed to address this symptom.

**Supplementary Information:**

The online version contains supplementary material available at 10.1186/s13023-024-03349-w.

## Background

Epidermolysis bullosa (EB) comprises a heterogeneous group of rare inherited mucocutaneous fragility disorders. The four major forms are determined by the ultrastructural level of blistering at the basement membrane zone (BMZ): EB simplex (EBS), junctional EB (JEB), dystrophic EB (DEB), and Kindler EB (KEB) [1]. Recessive DEB (RDEB) results from biallelic mutations in the type Vll collagen gene, *COL7A1*, with subtypes defined by molecular and clinical features, specifically severe (RDEB-S), intermediate (RDEB-I), inversa (RDEB-Inv), pruriginosa (RDEB-Pru) and localised (RDEB-L) forms [1]. RDEB-Inv and RDEB-Pru are frequently diagnosed later in life when specific features manifest. Although phenotypic features and severity vary across these subtypes, all are characterized by blisters and wounds which heal with scarring of skin and mucosae, leading to sequelae such as acral and joint contractures, corneal and oral mucosal scarring, and oesophageal and urethral strictures. In addition, there is an increased incidence of aggressive mucocutaneous squamous cell carcinomas (SCC) from early adulthood on [1].

Prevalence estimates for all forms of EB vary from 11.1/million in the USA [2] to 22.4/million in the Netherlands [3] and 34.8/million in England and Wales [4]. For RDEB specifically, estimated prevalence in the UK is 1.4–3.3/million with incidence of 3.05–8.1/million live births [4]. Although novel translational therapies including gene, protein and cell therapy as well as drug repurposing have become the focus for preclinical studies and clinical trials, current treatment for EB remains supportive rather than curative.

Pain, both nociceptive and neuropathic, arising from chronic cutaneous injury, is ubiquitous in all forms of EB [5–8], stemming from various sources but primarily due to cutaneous blisters and wounds which are often chronic and infected, impeding healing and exacerbating pain [9, 10]. Neonates with RDEB are frequently born with cutaneous damage or sustain wounding in the early days of life resulting in very early onset pain [10]. Pain also occurs from corneal abrasions, oral ulceration, dental caries, oesophageal strictures and reflux, constipation, anal fissures, joint contractures, osteoporosis and crush fractures [9, 11, 12]. SCC, a later complication, most notably in RDEB-S, is a further source of pain [13]. In addition, surgical procedures such as oesophageal dilatation, release of hand contractures and cancer surgery cause acute pain [10].

Background pain is a constant feature for many individuals with RDEB and can be intractable [5]. Pain is exacerbated by bathing and dressing changes, and frequently compromises activities of daily living (ADL), leading to sleep disruption, and restricted mobility and leisure activities, which all impact negatively on quality of life (QOL) [5, 8]. Psychological ‘pain’, the anxiety and emotional distress of living with EB, impacts on perceptions of symptoms and the ability to endure [14, 15]. While a combination of strategies for pain relief is common in RDEB, including conventional, psychological and less common therapies [10], potential nervous system sensitisation and psychological perspectives may limit effectiveness [16].

The Prospective Epidermolysis Bullosa Longitudinal Evaluation Study (PEBLES) is a prospective register study designed to delineate the natural history of different subtypes of RDEB throughout all ages from birth to death. Regular participant reviews build a comprehensive overview of specific health issues in RDEB, including severity scores, patient/family-reported outcomes, and detailed health economic data. Findings will help prognostication, inform outcome measures, and serve as proxy control data for future clinical trials. Here, we report PEBLES findings regarding background and procedural pain intensity for adults and children with different subtypes of RDEB, sleep disturbance due to pain, location of pain and medications used to manage pain. We also explore how quality of life and disease severity correlate with reported pain.

## Methods

### Study population

Individuals with RDEB attending the London EB centres (Great Ormond Street Hospital (children), Guy’s and St Thomas’ Hospital (adults)) were recruited to PEBLES over an 8-year period (November 2014 - September 2022). RDEB diagnosis was confirmed by skin biopsy and/or genetic testing, with subtype determined by clinical features and skin immunofluorescence findings where appropriate. The same data were collected at initial review and each subsequent review, undertaken 6-monthly in under-10s and annually for those aged 10 years and older, to capture information on EB- and non-EB-related health issues, disease severity and impact, and treatment received. Data were pseudonymised (date of birth retained to link participants’ age to reviews) and recorded in a Research Electronic Data Capture (REDCap) database. PEBLES was ethically approved by the UK Research Ethics Committee and Health Research Authority (IRAS 142032).

### Measures

All participants recorded average background and procedural pain in the preceding month using a 100 mm visual analogue scale (VAS), and answered questions (ordinal-level data) regarding location and intensity of pain and number of nights when sleep was disturbed by pain. Participants were asked about pain medications taken regularly and as required; these were categorised as strong or weak opioids, non-opioids (including non-steroidal anti-inflammatory drugs (NSAIDs) and paracetamol) and adjunctive pain medication such as antidepressants.

Symptom severity was recorded using two validated tools: the Birmingham Epidermolysis Bullosa Severity score (BEBS) [17], with clinician assessment scored out of a maximum of 100, and the two-part instrument for scoring clinical outcomes of research for epidermolysis bullosa (iscorEB) with a clinician assessment (ISC) scored out of a maximum of 138 and self-reported symptoms and disease impact (ISP), including 5 items about pain, scored out of 120 [18]. Skin involvement and wounding scores were reported by clinicians in both tools and separately considered. Participants also completed an age-appropriate QOL tool which included a single item about pain: QOLEB (adults) [19] or PedsQL (2–17 years) [20].

### Statistical analysis

Continuous variables are summarised using medians and interquartile range (IQR), and categorical variables using counts and percentages. Findings are presented for the RDEB cohort as a whole and by RDEB subtype at their index visit and as an average of per-participant metrics from all available reviews; data for the sole participant with pretibial RDEB (RDEB-PT) were included in the overall analysis but were excluded from subtype analysis. The index visit was the first available review with complete VAS pain metrics *and* complete data for the 5 pain-related questions within iscorEB; one adult participant with RDEB-S lacked a complete index review because iscorEB pain data were provided but no VAS pain metrics. Fifteen reviews (from 14 individuals) were excluded as lacking sufficient pain data for analysis. Otherwise, missing data are reported where relevant in the tables and figures.

Procedural pain VAS are reported only for participants with regular dressing changes at the time of review. Comparisons between RDEB subtypes for the different parameters of pain and RDEB severity at index review were computed using the Mann-Whitney U test with p-values adjusted using the Benjamini-Hochberg procedure; only the index review was considered as the test assumes observations are independent. All participants with RDEB-S were included in a linear mixed model that considered the outcomes of background and procedural pain (VAS) adjusted for age and the BEBS total score (chosen because BEBS had fewer missing scores than other severity scores).

Correlations and 95% confidence intervals (CI) were calculated using Spearman’s rank correlation. We used Cohen’s (1988) suggestion for interpreting correlation coefficients as: small, *r* = .10–0.29; medium, *r* = .30–0.49; large, *r* = .50-1.0. We defined statistical significance as *p* < .05. All analysis was performed using R (v4.1.3).

## Results

Pain scores were available for 61 participants who provided 361 reviews, including 25 individuals with RDEB-S (175 reviews), 22 with RDEB-I (108 reviews), 9 with RDEB-Inv (56 reviews), 4 with RDEB-Pru (17 reviews), and 1 with RDEB-PT (5 reviews). Table 1 shows participant demographics at index review.

Disease severity scores (iscorEB, BEBS) at index review were higher for participants with RDEB-S and RDEB-Pru than those with intermediate and inversa subtypes (Table 2). Similarly, severe and pruriginosa participants had higher QOLEB scores (indicating greater negative impact on QOL) and spent more time on dressing changes than the other subtypes (Table 2). Similar findings were revealed on consideration of all 361 reviews (Supplementary Table 1).

**Table 1:** Participant characteristics by RDEB subtype (*n* = 61)

Characteristic	Category	Overall	RDEB-S	RDEB- I	RDEB-Inv	RDEB-PT	RDEB-Pru
n		61	25	22	9	1	4
Age group (years)	0 < 10	10 (16)	8 (32)	2 (9)	0 (0)	0 (0)	0 (0)
	10 < 18	3 (5)	2 (8)	1 (5)	0 (0)	0 (0)	0 (0)
	18 < 40	23 (38)	12 (48)	5 (23)	5 (56)	0 (0)	1 (25)
	≥ 40	25 (41)	3 (12)	14 (64)	4 (44)	1 (100)	3 (75)
Age (years)		34 [22,49]	23 [8,33]	47 [32,63]	39 [30,48]	72 [72,72]	49 [40,57]
Gender	Female	34 (56)	13 (52)	14 (64)	6 (67)	0 (0)	1 (25)
	Male	27 (44)	12 (48)	8 (36)	3 (33)	1 (100)	3 (75)
Ethnicity	White	51 (84)	18 (72)	20 (91)	8 (89)	1 (100)	4 (100)
	Asian	7 (11)	5 (20)	1 (5)	1 (11)	0 (0)	0 (0)
	Black	0 (0)	0 (0)	0 (0)	0 (0)	0 (0)	0 (0)
	Mixed	2 (3)	2 (8)	0 (0)	0 (0)	0 (0)	0 (0)
	Other	1 (2)	0 (0)	1 (5)	0 (0)	0 (0)	0 (0)
Participant employment	Employed (Full/part time)	19 (31)	3 (12)	9 (41)	5 (56)	0 (0)	2 (50)
	Unemployed	17 (28)	8 (32)	3 (14)	4 (44)	0 (0)	2 (50)
	Retired	7 (11)	0 (0)	6 (27)	0 (0)	1 (100)	0 (0)
	N/A (child/higher education)	18 (30)	14 (56)	4 (18)	0 (0)	0 (0)	0 (0)
Parent employment	Employed (Full/part time)	14 (23)	10 (40)	3 (14)	0 (0)	0 (0)	0 (0)
Number of reviews, n		6 [4,7]	7 [5,8]	6 [3,7]	7 [6,7]	5 [5,5]	4 [2,6]
Period of reviews (years)		6 [3,7]	6 [5,7]	6 [2,6]	6 [6,7]	4 [4,4]	4 [2,7]

Results presented as n(%) or median [IQR]

**Table 2:** RDEB severity at index review (*n* = 61)

Severity scores	Overall	RDEB-S	RDEB-I	RDEB-Inv	RDEB-Pru
n	61	25	22	9	4
ISC total score^1^	66 [42,81] (*n* = 56)	76 [66,102] (*n* = 23)	54 [26,71] (*n* = 19)	42 [37,59]	84 [58,99]
ISC clinician score^2^	19 [7,30] (*n* = 56)	30 [21,40] (*n* = 23)	10 [6,18] (*n* = 19)	6 [5,7]	22 [15,28]
ISC patient score^3^	44 [28,57] (*n* = 60)	48 [41,61] (*n* = 23)	28 [14,51]	37 [30,54]	57 [43,66]
BEBS total score^4^	26 [11,38]	40 [29,46] (*n* = 24)	15 [6,24]	9 [8,14]	23 [20,29]
ISC skin score^5^	8 [2,15] (*n* = 57)	15 [12,21] (*n* = 23)	3 [1,7] (*n* = 21)	2 [0,3]	10 [6,14]
BEBS skin score^6^	8 [2,15]	16 [12,22] (*n* = 24)	3 [1,6]	1 [0,1]	11 [8,18]
QOLEB total score^7^ (adults only)	20 [13,28] (*n* = 46)	23 [20,32] (*n* = 16)	14 [8,23] (*n* = 18)	17 [13,22] (*n* = 7)	30 [25,32] (*n* = 4)
PedsQL total, ^8^ parent score	44 [39,51] (*n* = 8)	47 [40,52] (*n* = 7)	38 [38,38] (*n* = 1)		
PedsQL total, ^8^ patient score	52 [47,56] (*n* = 7)	50 [46,56] (*n* = 6)	54 [54,54] (*n* = 1)		
Annual dressing time, hrs	364 [91,585] (*n* = 53)	585 [351,910] (*n* = 25)	61 [30,364] (*n* = 20)	121 [67,121] (*n* = 3)	442 [281,815] (*n* = 3)
*Dressing frequency*					
• All at once	48 (79)	20 (80)	20 (91)	3 (33)	4 (100)
• Few at a time	6 (10)	5 (20)	0 (0)	1 (11)	0 (0)
• None required	6 (10)	0 (0)	1 (5)	5 (56)	0 (0)
• Infrequent	1 (2)	0 (0)	1 (5)	0 (0)	0 (0)

^1^ Total of iscorEB clinician and patient scores, maximum of 258

^2^ iscorEB clinician score, maximum of 138

^3^ iscorEB patient score, maximum of 120

^4^ BEBS, Birmingham EB Severity score, maximum of 100

^5^ Component of iscorEB clinician score, maximum of 78

^6^ Component of BEBS, maximum of 50

^7^ QOLEB, Quality of Life in Epidermolysis Bullosa questionnaire, maximum of 51

^8^ PedsQL, Pediatric Quality of Life Inventory, maximum of 100; higher score = lesser severity

Results presented as n(%) or median [IQR] with participant numbers reported where results related to only some of the group

### Intensity of background and procedural pain

Most participants (93%) reported pain at index review (Table 3), including all those with RDEB-S and RDEB-Pru. Fifty individuals (80%) reported both background and procedural pain. Only three participants with RDEB-I (2 adults, 1 child) reported no background pain at all reviews (*n* = 13). Another 10 adults and 4 children under 10 years reported background pain at some reviews and not at others, including 2 adults and 3 children with RDEB-S (8 reviews), 6 adults and 1 child with RDEB-I (19 reviews), and 2 adults with RDEB-Inv (2 reviews).

**Table 3:** Background and procedural pain VAS by RDEB subtype at index review (*n* = 61)

Variable	Overall	RDEB-S	RDEB-I	RDEB-Inv	RDEB-Pru
n	61	25	22	9	4
Background and/or procedural pain > 0 mm on VAS	57 (93)	25 (100)	19 (86)	8 (89)	4 (100)
Background pain VAS	40 [20,60]	39 [20,54]	40 [12,69]	30 [30,40]	58 [41,62]
Procedural pain VAS^1^	52 [40,80] (*n* = 54)	60 [40,75]	45 [35,72] (*n* = 20)	40 [22,54] (*n* = 4)	82 [62,86]
Difference between procedural and background pain VAS (Procedural – Background pain)	10 [0,21] (*n* = 54)	20 [10,30]	0 [-1,20] (*n* = 20)	0 [-3,2] (*n* = 4)	22 [18,25]

VAS, visual analogue scale measured from 0–100 mm

Results are presented as n (%) or median [IQR] (n)

Participant numbers reported where results related to only some of the group

^1^Excludes those report no/infrequent dressing changes

**Fig. 1:**
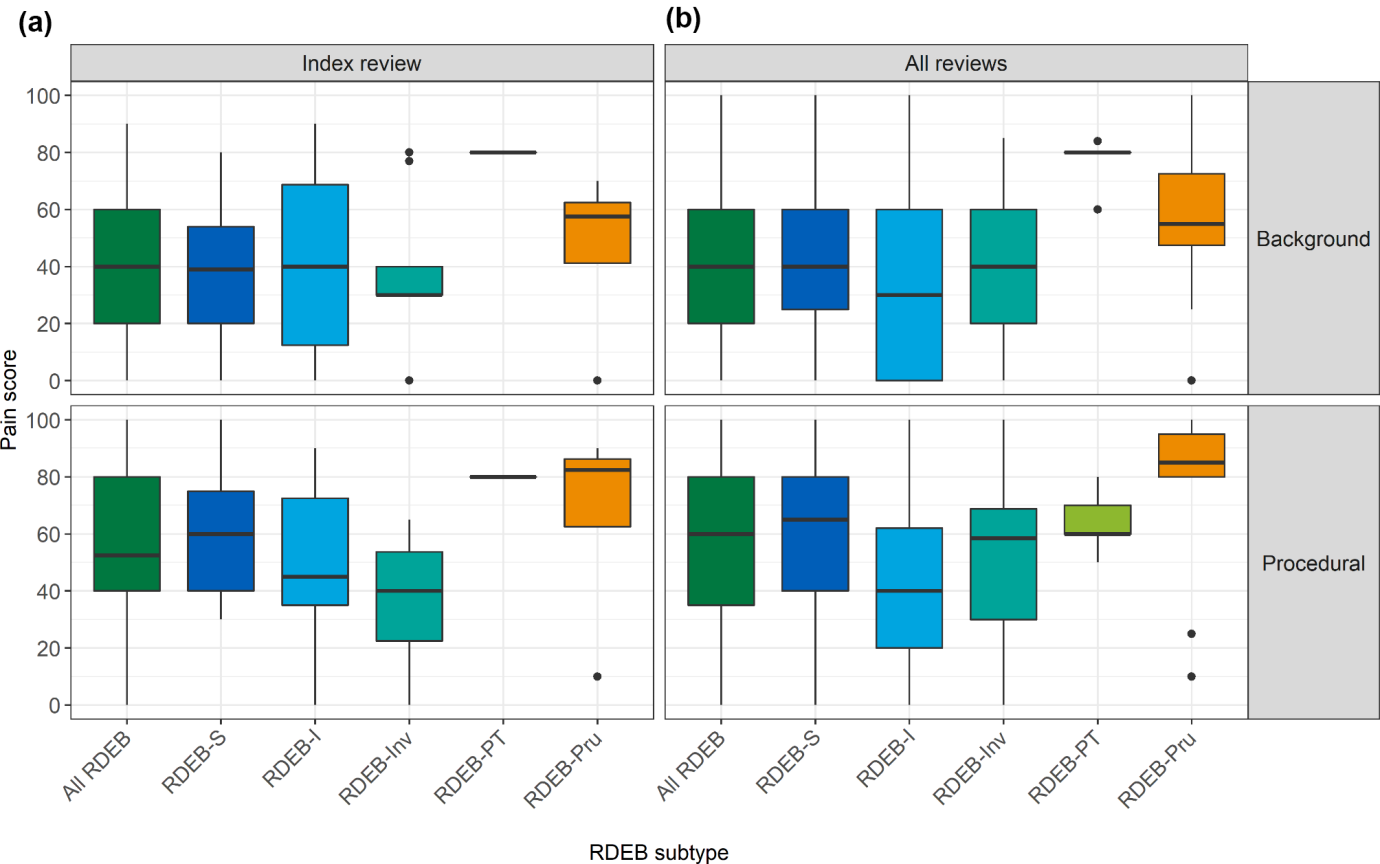
Box and whisker plot of background and procedural pain VAS at (**a**) index review (*n* = 61) and (**b**) all reviews (*n* = 361). Procedural pain VAS (*n* = 54) excluded participants reporting no/infrequent frequent dressing changes. Data reported in Table 3 and Supplementary Table 2

Median background pain VAS at index review for all RDEB was 40 [20,60] out of 100, with RDEB-Pru reporting the greatest pain (Table 3; Fig. 1), and a similar pattern of background pain when all reviews were considered (Fig. 1, Supplementary Table 2). Background pain VAS scores at index review were positively associated with severity scores for all RDEB with medium to large effect size (Table 4). When considering all reviews, background pain VAS scores for all RDEB and for RDEB-I were moderate or strongly positively associated with iscorEB and BEBS severity scores, and weakly correlated for other subtypes (Supplementary Table 3).

**Table 4:** Correlations between VAS background pain scores and severity scores by subtype at index review (*n* = 61)

Variable 1	Variable 2	Overall	RDEB-S	RDEB-I	RDEB-Inv	RDEB-Pru
Background pain VAS^*1*^	iscorEB clinician score^*3*^	*0.32* *[0.06*,*0.53]* (*n* = 56)	0.29 [-0.14,0.63] (*n* = 23)	**0.61** **[0.22**,**0.83]** (*n* = 19)	0.37 [-0.39,0.83] (*n* = 9)	1.00 [1.00,1.00] (*n* = 4)
ISP overall pain^*2*^	iscorEB clinician score^*3*^	*0.44* *[0.20*,*0.63]* (*n* = 54)	**0.53** **[0.14**,**0.78]** (*n* = 22)	**0.61** **[0.20**,**0.84]** (*n* = 18)	0.23 [-0.51,0.78] (*n* = 9)	0.95 [-0.14,1.00] (*n* = 4)
Background pain VAS^*1*^	iscorEB patient score^*4*^	**0.71** **[0.56**,**0.82]** (*n* = 61)	*0.46* *[0.08*,*0.72]* (*n* = 25)	**0.89** **[0.75**,**0.95]** (*n* = 22)	0.86 [0.45,0.97] (*n* = 9)	0.80 [-0.70,1.00] (*n* = 4)
ISP overall pain^*2*^	iscorEB patient score^*4*^	**0.82** **[0.71**,**0.89]** (*n* = 59)	**0.68** **[0.38**,**0.85]** (*n* = 24)	**0.88** **[0.72**,**0.95]** (*n* = 21)	0.83 [0.36,0.96] (*n* = 9)	0.95 [-0.14,1.00] (*n* = 4)
Background pain VAS^*1*^	iscorEB total score^*5*^	**0.61** **[0.41**,**0.75]** (*n* = 56)	**0.55** **[0.18**,**0.79]** (*n* = 23)	**0.85** **[0.64**,**0.94]** (*n* = 19)	0.86 [0.45,0.97] (*n* = 9)	0.80 [-0.70,1.00] (*n* = 4)
ISP overall pain^*2*^	iscorEB total score^*5*^	**0.70** **[0.53**,**0.81]** (*n* = 54)	**0.70** **[0.40**,**0.87]** (*n* = 22)	**0.80** **[0.53**,**0.92]** (*n* = 18)	0.80 [0.29,0.96] (*n* = 9)	0.95 [-0.14,1.00] (*n* = 4)
Background pain VAS^*1*^	iscorEB skin score^*6*^	*0.35* *[0.10*,*0.56]* (*n* = 58)	*0.35* *[-0.07*,*0.67]* (*n* = 23)	**0.61** **[0.25**,**0.83]** (*n* = 21)	-0.24 [-0.78,0.51] (*n* = 9)	1.00 [1.00,1.00] (*n* = 4)
ISP overall pain^*2*^	iscorEB skin score^*6*^	*0.45* *[0.22*,*0.64]* (*n* = 58)	*0.40* *[-0.01*,*0.70]* (*n* = 23)	*0.49* *[0.08*,*0.76]* (*n* = 21)	-0.09 [-0.71,0.61] (*n* = 9)	0.95 [-0.14,1.00] (*n* = 4)
Background pain VAS^*1*^	BEBS total score^*7*^	*0.33* *[0.08*,*0.54]* (*n* = 60)	0.29 [-0.13,0.62] (*n* = 24)	**0.68** **[0.37**,**0.86]** (*n* = 22)	0.31 [-0.45,0.81] (*n* = 9)	1.00 [1.00,1.00] (*n* = 4)
ISP overall pain^*2*^	BEBS total score^*7*^	*0.44* *[0.21*,*0.63]* (*n* = 58)	*0.34* *[-0.08*,*0.66]* (*n* = 23)	**0.66** **[0.32**,**0.85]** (*n* = 21)	0.17 [-0.56,0.75] (*n* = 9)	0.95 [-0.14,1.00] (*n* = 4)
Background pain VAS^*1*^	BEBS skin score^*8*^	*0.32* *[0.07*,*0.53]* (*n* = 60)	0.28 [-0.13,0.62] (*n* = 24)	**0.67** **[0.35**,**0.85]** (*n* = 22)	-0.24 [-0.78,0.51] (*n* = 9)	1.00 [1.00,1.00] (*n* = 4)
ISP overall pain^*2*^	BEBS skin score^*8*^	*0.43* *[0.20*,*0.62]* (*n* = 58)	*0.35* *[-0.07*,*0.67]* (*n* = 23)	**0.63** **[0.28**,**0.84]** (*n* = 21)	-0.27 [-0.79,0.48] (*n* = 9)	0.95 [-0.14,1.00] (*n* = 4)

Variable 1, Patient-reported pain scores:

^1^ VAS, visual analogue scale

^2^ Question 1 of iscorEB patient questionnaire

Variable 2, Clinician and patient-reported severity scores:

^3^ iscorEB clinician score

^4^ iscorEB patient score

^5^ Total of iscorEB clinician and patient scores

^6^ Component of iscorEB clinician score

^7^ BEBS, Birmingham EB Severity score

^8^ Component of BEBS

Results presented as correlation [95% CI] (n), calculated using Spearman’s rank correlation. Results are significant if 95% CI does not include 0; correlations where *n* < 10 should be considered with caution as associations could be spurious

Participant numbers reported where results related to only some of the group

Significant associations: **large** (bold text), *r* = .50 − 1.0; medium (italics), *r* = .30-0.49. Associations not highlighted in groups where *n* < 10

Median procedural pain VAS for participants reporting regular wound dressing changes at index review (*n* = 54) was 52 [40,80], which was a median 10 [0,21] points greater than reported background pain (Fig. 1; Table 3). At index review, individuals with RDEB-S and RDEB-Pru reported a distinct difference between procedural and background pain, 20 and 22 points, respectively, whereas those with RDEB-I and RDEB-Inv reported no difference (Table 3); the findings were similar when all reviews were considered (Supplementary Table 2).

Greater procedural pain at index review and when considering all reviews was positively associated with worse severity scores and longer time spent on dressing changes (Supplementary Tables 4 and 5). When considering subtype at index review, the only significant correlation was between procedural pain and iscorEB patient score (ISP) and iscorEB total score for RDEB-S and RDEB-I (Supplementary Table 4). For those with RDEB-S, pain was positively associated with BEBS scores with a 10-unit increase in BEBS increasing background pain by 5 points [95%CI: 1,9; *p* = .01] and procedural pain by 4 points [95%CI: 0,8; *p* = .04].

There was a large correlation between background and procedural pain at index review and adult QOLEB scores (and functioning and emotions subscores) for all subtypes except RDEB-Pru (Table 5). Similar results were observed for all reviews (Supplementary Tables 6 and 7). Thus, worse pain was associated with poorer QOL for adults. The relationship between parent and child QOL scores (PedsQL) and pain VAS varied widely and was difficult to interpret due to small review numbers (Supplementary Tables 6 and 7).

**Table 5:** Correlations between QOL and background and procedural pain scores by subtype at index review, adults only (*n* = 49)

Variable 1	Variable 2	Overall	RDEB-S	RDEB-I	RDEB-Inv	RDEB-Pru
QOLEB functioning score^*1*^	VAS Background pain	**0.55** **[0.31**,**0.72]** (*n* = 46)	**0.64** **[0.21**,**0.86]** (*n* = 16)	**0.71** **[0.37**,**0.89]** (*n* = 18)	0.87 [0.33,0.98] (*n* = 7)	0.80 [-0.70,1.00] (*n* = 4)
QOLEB emotions score^*2*^	VAS Background pain	**0.59** **[0.37**,**0.75]** (*n* = 49)	**0.54** **[0.07**,**0.82]** (*n* = 16)	**0.67** **[0.32**,**0.86]** (*n* = 19)	0.92 [0.66,0.98] (*n* = 9)	-0.26 [-0.98,0.93] (*n* = 4)
QOLEB total score^*3*^	VAS Background pain	**0.63** **[0.42**,**0.78]** (*n* = 46)	**0.74** **[0.38**,**0.90]** (*n* = 16)	**0.71** **[0.36**,**0.88]** (*n* = 18)	0.88 [0.39,0.98] (*n* = 7)	0.80 [-0.70,1.00] (*n* = 4)
QOLEB functioning score^*1*^	VAS Procedural pain	**0.54** **[0.28**,**0.73]** (*n* = 40)	**0.51** **[0.02**,**0.80]** (*n* = 16)	0.40 [-0.12,0.75] (*n* = 16)	n/a (*n* = 3)	0.80 [-0.70,1.00] (*n* = 4)
QOLEB emotions score^*2*^	VAS Procedural pain	**0.66** **[0.45**,**0.80]** (*n* = 42)	**0.65** **[0.23**,**0.87]** (*n* = 16)	**0.62** **[0.20**,**0.85]** (*n* = 17)	1.00 [1.00,1.00] (*n* = 4)	-0.26 [-0.98,0.93] (*n* = 4)
QOLEB total score^*3*^	VAS Procedural pain	**0.67** **[0.45**,**0.81]** (*n* = 40)	**0.66** **[0.24**,**0.87]** (*n* = 16)	0.46 [-0.04,0.78] (*n* = 16)	n/a (*n* = 3)	0.80 [-0.70,1.00] (*n* = 4)

Variable 1: QOLEB, Quality of Life in Epidermolysis Bullosa questionnaire scores

^1^ Subscore of QOLEB

^2^ Subscore of QOLEB

^3^ Total of QOLEB

Variable 2: Pain scores, VAS, visual analogue scale; only participants with frequent dressing changes were included in procedural pain correlations

Results presented as correlation [95% CI] (n), calculated using Spearman’s rank correlation

Results are significant if 95% CI does not include 0; correlations where *n* < 10 should be considered with caution as associations could be spurious

Significant associations: **large** (bold text), *r* = .50 − 1.0; medium (italics), *r* = .30-0.49. Associations not highlighted in groups where *n* < 10

There were too few participants to explore differences in pain according to age at index review. However, when considering all reviews for RDEB-S, children under 10 years reported less procedural pain and their difference between background and procedural pain was smaller than for all other age groups; older participants with RDEB-S reported some pain at all reviews, whereas 4 (7%) child reviews with RDEB-S reported no background or procedural pain.

### Frequency of pain

Half the participants, 27 (55%) adults and 5 (56%) children, reported pain as ‘frequent’/’often’ or ‘constant’/’always’ at index review (Table 6). All adults and children with RDEB-S reported pain, whereas other subtypes reported greater variation in pain frequency. Supplementary Table 8 shows a similar pattern for adults (55%) when considering all reviews, although slightly less frequency for children (44%).

**Table 6:** Frequency of reported pain at index review by RDEB subtype (*n* = 61)

Variable	Category	Overall	RDEB-S	RDEB-I	RDEB-Inv	RDEB-Pru
Weekly sleep disturbed pain	0 nights	21 (34)	5 (20)	12 (55)	3 (33)	1 (25)
	1–3 nights	17 (28)	10 (40)	4 (18)	3 (33)	0 (0)
	4–6 nights	10 (16)	4 (16)	3 (14)	0 (0)	3 (75)
	Every night	13 (21)	6 (24)	3 (14)	3 (33)	0 (0)
Does EB cause physical pain? (QOLEB Q3)^1^	No pain	5 (10)	0 (0)	3 (16)	1 (11)	1 (25)
	Occasional pain	17 (35)	7 (44)	8 (42)	2 (22)	0 (0)
	Frequent pain	15 (31)	5 (31)	6 (32)	4 (44)	0 (0)
	Constant pain	12 (24)	4 (25)	2 (11)	2 (22)	3 (75)
Do you have aches and pains? (PedsQL parent)^2^	Never	0 (0)	0 (0)	0 (0)		
	Almost never	1 (11)	1 (12)	0 (0)		
	Sometimes	3 (33)	3 (38)	0 (0)		
	Often	4 (44)	3 (38)	1 (100)		
	Almost always	1 (11)	1 (12)	0 (0)		
Do you have aches and pains? (PedsQL patient)^3^	Never	0 (0)	0 (0)	0 (0)		
	Almost never	0 (0)	0 (0)	0 (0)		
	Sometimes	3 (43)	3 (50)	0 (0)		
	Often	1 (14)	0 (0)	1 (100)		
	Almost always	3 (43)	3 (50)	0 (0)		

^1^ Adults only, *n* = 49; RDEB-S = 16, RDEB-I = 19, RDEB-Inv = 9, RDEB-PR = 4

^2^ Parents of child participants aged 2-18y, *n* = 9; RDEB-S = 8, RDEB-I = 1. Two children with RDEB-I were aged < 2y so too young to complete PedsQL. RDEB-S = 1 parent score missing

^3^ Child participants aged 5-18y, *n* = 7; RDEB = 6, RDEB-I = 1. Another 5 children < 5y so too young to complete PedsQL

One third of participants (38% index review, 37% all reviews) reported at least 4 nights disturbed sleep each week due to pain, with RDEB-Pru reporting the greatest disturbance (75% index review, 88% all reviews) (Table 6, Supplementary Table 8). However, one third of all RDEB reported no sleep disturbance (34% index, 34% all reviews), with a greater number of individuals with RDEB-I reporting no sleep disturbance in the previous month (55% index, 56% all reviews).

### Location and intensity of pain

**Fig. 2:**
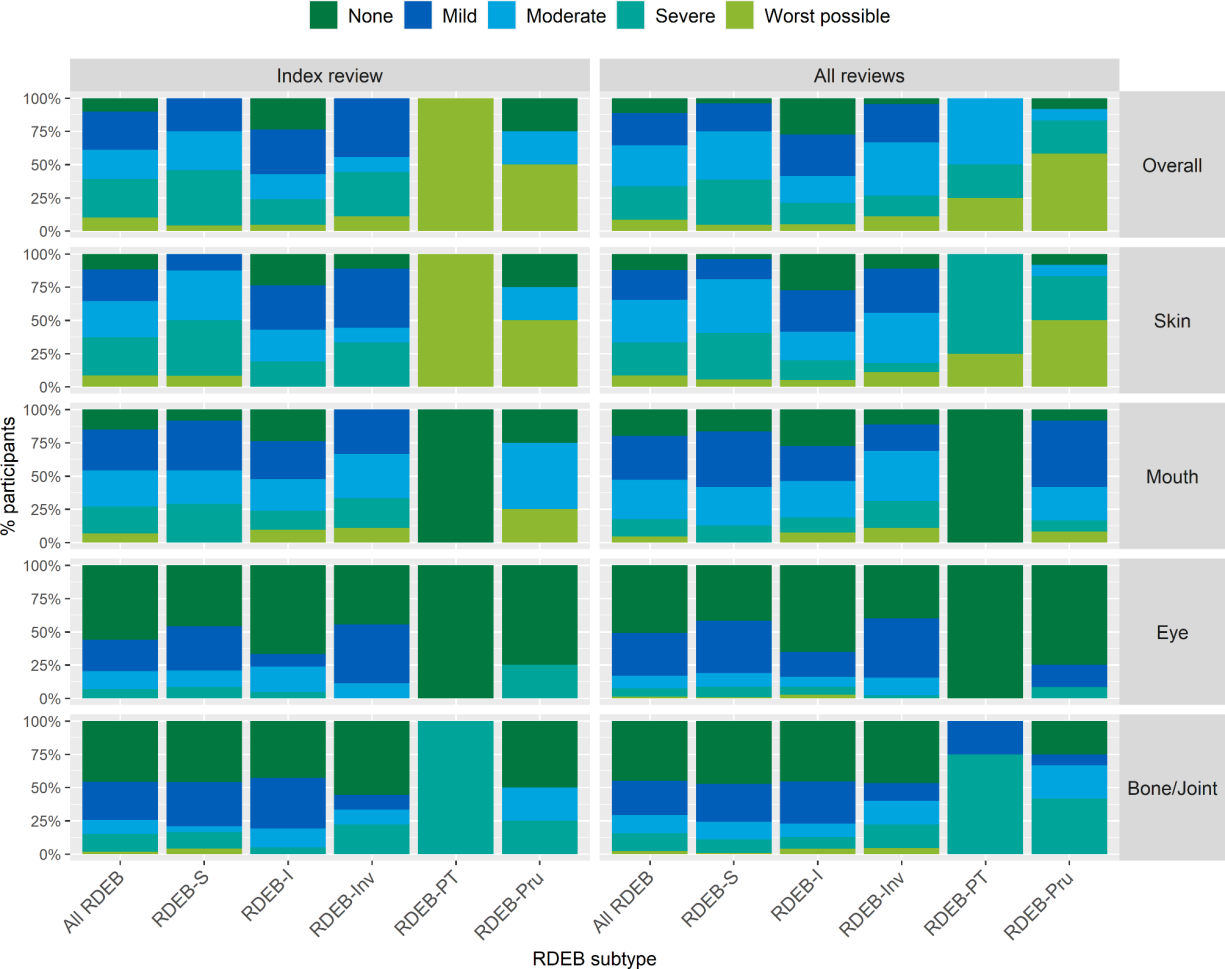
Location and intensity of pain reported on iscorEB patient questionnaire when considering all reviews (*n* = 268). Data reported in Table 3 and Supplementary Table 9. Findings for index reviews cannot be reported as these early reviews retained only manual sub-scores so individual item scores not available for these

Figure 2 shows the variation in pain location and intensity when considering all reviews reported by different RDEB subtypes, with variation within and between subtypes (see also Supplementary Table 9). The most reported pain location was the skin, except for RDEB-Inv where mouth pain was more problematic. Individuals with RDEB-I reported lower pain frequency and intensity for each location, whereas those with RDEB-Pru reported greater intensity of overall pain, skin and bone/joints pain, although numbers were small and pain location did not correlate with any severity metrics. When outcomes were compared using the Mann-Whitney U test, with P-values adjusted using the Benjamini-Hochberg procedure, the only significant difference in pain location was between RDEB-S and RDEB-I, *p* = .026.

Moderate-large correlations for all RDEB were found between reported skin pain and BEBS score, likely due to severity of skin wounding (BEBS skin score), and dressing time (Supplementary Tables 10 and 11). Surprisingly, while there were significant correlations for milder subtypes (RDEB-I and RDEB-Inv), there were no significant correlations for those subtypes with greater wounding (RDEB-S and RDEB-Pru).

### Treatment of pain

**Fig. 3:**
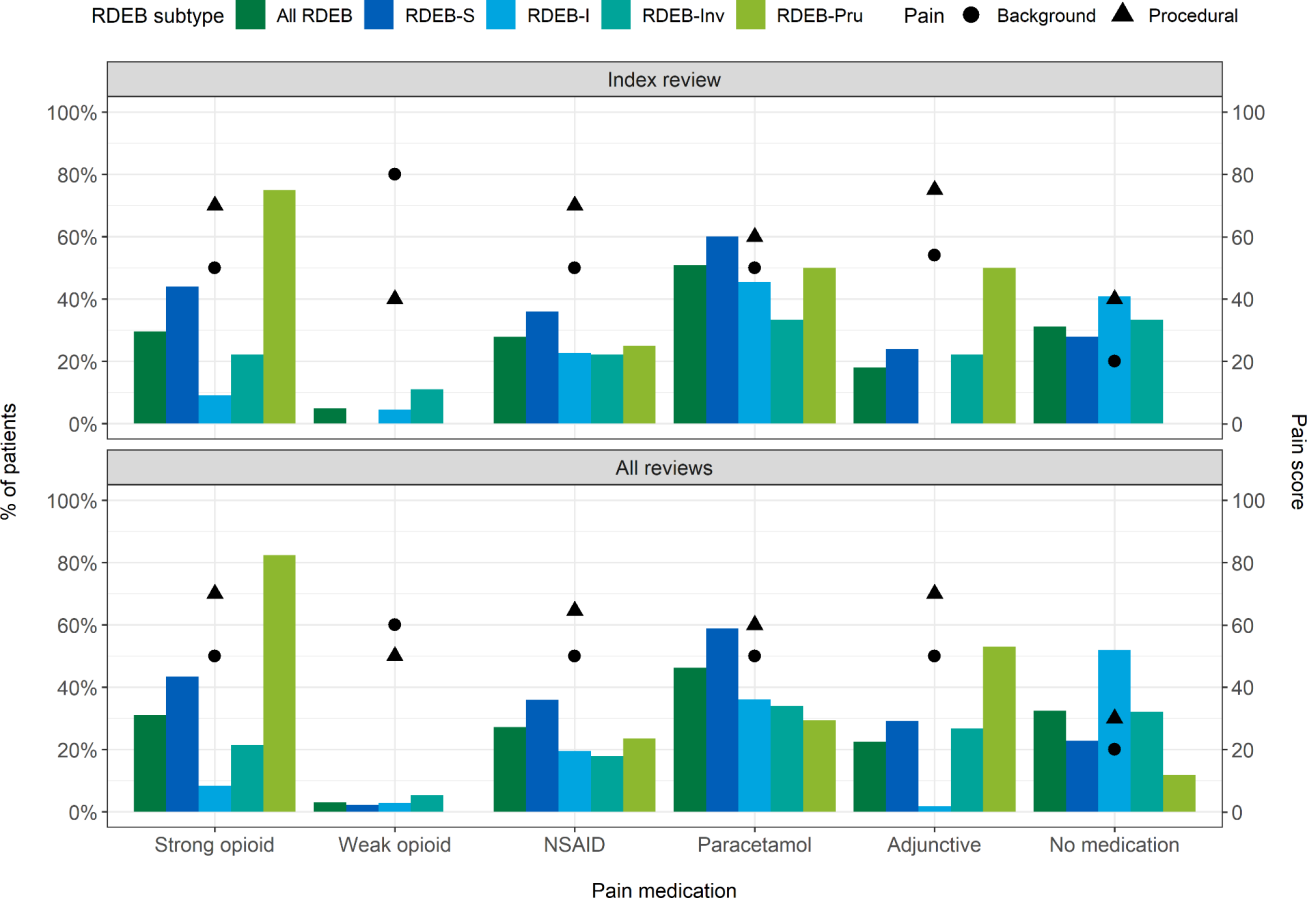
Pain medication usage at index review (*n* = 61) and all reviews (*n* = 361). Some participants reported using more than one type of medication. Data reported in Supplementary Table 12; Pain VAS, visual analogue scale measured from 0–100 mm

One third of participants (31%) reported no pain medication usage at index review. Another 31% used regular and/or ‘as required’ (PRN) medication and 38% reported only PRN medication. Figure 3 (data in Supplementary Table 12) shows similar findings for median background and procedural pain VAS scores at index review and when considering all reviews for participants using different types of pain medication; many participants recorded more than one type of medication so may be reported more than once. Individuals of all subtypes using regular and/or PRN medication reported higher background and procedural pain VAS scores than those reporting only PRN medication (Supplementary Table 13). Individuals who did not report use of pain medication were more likely to report infrequent/no dressing changes than those using pain medication (21% vs. 7%) and also reported less annual dressing time (91 vs. 364 h), although these differences were not statistically significant. RDEB-S and RDEB-Pru reported the greatest strong opioid usage at index and all reviews (Supplementary Table 13).

## Discussion

Pain is often cited as a major problem in EB and is an area patients and caregivers have identified as a priority for better treatments [8, 21]. However, the incidence, intensity, sites, and frequency of pain have not been comprehensively explored and most studies have reported global pain in all EB [8, 16], all DEB [22, 23] or all RDEB [7, 24]. Ours is the first study to report pain in detail by RDEB subtype.

There are many different potential sources of pain in EB (e.g. inflammatory and neuropathic skin pain, corneal abrasions, musculoskeletal contractures, dental pain etc.), yet only one study has explored skin pain specifically [5]. Despite the burden of frequent dressing changes in most individuals with RDEB, procedural pain specifically has only been reported in one study rating acute and chronic pain levels in individuals with EB [25]. Given the clinical heterogeneity of different forms of EB in general and within RDEB, specifically, we consider reporting pain by subtype is essential to better understand this important symptom.

Fine et al. [5] reported the highest cutaneous pain scores in individuals with RDEB-S having the most extensive skin involvement. Similarly, another study found the highest acute pain scores were in RDEB-S, with greatest chronic pain equally in JEB and all RDEB [25]. However, another study of all RDEB [24] found no difference in pain scores across those self-reporting mild, moderate or severe disease severity, and a further study [26] found similar pain levels between RDEB-S and RDEB-I. In our study, high severity scores (BEBS, iscorEB) for all reviews correlated with higher background pain scores, most notably for RDEB-S and RDEB-I, and all RDEB-S participants reported pain. As would be expected due to more extensive skin damage, participants with RDEB-S and RDEB-Pru, the more severe subtypes, reported higher background pain scores than did milder subtypes, although we were unable to reliably report any other correlations due to small numbers for some subtypes.

Our findings for median 40 [20, 60] background pain scores (VAS 0-100) for all RDEB (at index and all reviews) are comparable with previous studies reporting pain in all RDEB subtypes using a VAS of 0–10, with those pain scores ranging from a mean (SD) of 4.2 (0.52) [7] to 5.4 [16], 6.54 (1.65) in a group of RDEB-S and RDEB-I [26], and a chronic pain score for all RDEB of 5.3 [25]. With regards to procedural pain, Bruckner et al. [25] reported acute pain in all RDEB as a mean of 6.4 (out of 10) whilst we report a slightly lower procedural pain score of 52 [40,80] (out of 100) across the 52 participants who reported regular dressing changes. The same group also found higher acute pain scores in those with the most severe and extensive skin damage who also had the longest dressing change durations [25], which is consistent with our results where participants with the more severe forms reported higher procedural pain levels. Our findings show that, while those with the most frequent and longer duration of dressing change used the most PRN analgesia, of note, the biggest difference between background and procedural pain scores are found in individuals with higher severity scores and lengthy dressing changes; this suggests background pain for this group may be partially controlled but procedural pain is not.

Over half of all PEBLES participants described frequent or constant pain whereas Bruckner described 15.9% with frequent and 22.2% with constant pain with the difference likely due to their inclusion of milder non-RDEB types of EB [25]. We found that worse pain negatively impacted QOL and sleep in the adult group but due to small numbers we are unable to report any association in children. Over one third of our participants reported sleep disturbed by pain on at least 4 nights of the week, which was greatest in RDEB-Pru (75% index reviews), albeit participant numbers were low, and possibly due to nocturnal itch exacerbating pain by destructive scratching causing skin damage [27].

Anatomical locations of pain were previously reported for a mixed group of all EB types who experienced pain (*n* = 39) where the most frequently reported site was the hands and feet [16] which was unsurprising as half the group (*n* = 19) had EBS whose effects are seen primarily in these areas. However, our study used iscorEB to locate pain sites by organ/tissue rather than anatomical location and, in descending order of frequency, participants identified the skin, mouth, eye and bone as sites of pain. Only in RDEB-Inv was mouth pain the commonest site of pain, as frequently oral tissues are one of the most affected areas in this subtype. As our data did not detail anatomical location of pain further, we were unable to comment on areas associated with particular impact such as genital or perianal pain which might also disproportionately affect individuals with RDEB-Inv. Interestingly, although skin pain correlated strongly with BEBS score across all RDEB and subtypes RDEB-I and RDEB-Inv, this was not the case for RDEB-S and RDEB-Pru groups, perhaps suggesting that skin pain is disproportionately higher in these types and may not be predicted from an objective severity tool.

We found that strong opioids were mainly used by those with the most extensive skin damage (RDEB-S, RDEB-Pru) which is in keeping with a previous study where disease severity was associated with increased opioid use [28]. However, we found these groups also reported the highest pain scores suggesting that, especially for procedural pain where the differences from background pain were greatest, treatment is at least partially ineffective. Our study did not differentiate between nociceptive and neuropathic pain but this would be an interesting area for further study given the evidence for small nerve fibre damage in the aetiology of RDEB pain [7]. The presence of both mechanisms of pain might go some way to explain the relative lack of efficacy of analgesic medication in RDEB demonstrated by our findings which underscore the unmet need for effective pain management especially for the more severe RDEB subtypes.

A strength of our study is the reporting by subtype that includes several reviews for individuals with RDEB, and the use of validated disease severity scores (BEBS, iscorEB). Limitations include the relative under-representation of children and of rarer subtypes, particularly RDEB-Pru and RDEB-PT. Pain medication was reported at time of review so does not reflect any changes in medication in the previous months, whereas participants were asked to report pain VAS as an average for the previous month. None of the tools ask about the effectiveness of pain medication so we do not know the impact on differences in reporting pain. We did not enquire about the timing of medications relative to procedures such as dressing changes; medication taken pre-emptively, before a procedure, would likely reduce the levels of pain experienced, whereas, if taken as needed during the procedure, pain may have been more intense up until that point.

## Conclusions

Our study, which specifically addresses pain in detail by RDEB subtype, highlights that pain is an almost universal symptom across all types of RDEB and is especially severe for those with RDEB-S and RDEB-Pru subtypes, and generally correlates with worse quality of life, greater disease severity and longer time spent on dressing changes. Procedural pain in particular appears poorly controlled, even by those using regular and as required medication including strong opioids. The one third of participants reporting no pain medication use, despite over 90% of all participants experiencing some pain, suggests that current treatments are inadequate and/or not tolerated; this indicates an unmet need for better therapies to address EB-related pain.

## Electronic supplementary material

Below is the link to the electronic supplementary material.

Supplementary Material 1

Supplementary Material 2

Supplementary Material 3

Supplementary Material 4

Supplementary Material 5

Supplementary Material 6

Supplementary Material 7

Supplementary Material 8

Supplementary Material 9

Supplementary Material 10

Supplementary Material 11

Supplementary Material 12

Supplementary Material 13

## Data Availability

The datasets generated and analysed during the current study are not publicly available as the authors intend to prepare further publications from them. However, the authors would consider reasonable requests to access the data and will make these available in an accessible repository once all relevant data have been published.

## Abbreviations

BEBS: Birmingham EB Severity (score)
EB: Epidermolysis Bullosa
IQR: Interquartile Range
ISC: IscorEB Clinician Score
iscorEB: Instrument for Scoring Clinical Outcomes of Research for EB
ISP: iscorEB Patient Score
QOL: Quality of Life
QOLEB: Quality of Life in EB questionnaire
PedsQL: Pediatric Quality of Life Inventory
RDEB: Recessive Dystrophic Epidermolysis Bullosa
RDEB-I: Intermediate Recessive Dystrophic Epidermolysis Bullosa
RDEB-Inv: Inversa Recessive Dystrophic Epidermolysis Bullosa
RDEB-Pru: Pruriginosa Recessive Dystrophic Epidermolysis Bullosa
RDEB-PT: Pretibial Recessive Dystrophic Epidermolysis Bullosa
RDEB-S: Severe Recessive Dystrophic Epidermolysis Bullosa
SCC: Squamous Cell Carcinoma
VAS: Visual Analogue Scale
