# Supplementary material for: Pain in recessive dystrophic epidermolysis bullosa (RDEB): findings of the Prospective Epidermolysis Bullosa Longitudinal Evaluation Study (PEBLES)
Source: Orphanet J Rare Dis. 2024 Oct 11;19:375. doi: 10.1186/s13023-024-03349-w (PMC11468479; doi:10.1186/s13023-024-03349-w)
Supplement: Supplementary file 1 — Supplementary Material 1 [file 13023_2024_3349_MOESM1_ESM.docx]

**Supplementary Table 1. Severity scores for all reviews (n=361).**

| Variable | Overall | RDEB-S | RDEB-I | RDEB-Inv | RDEB-Pru |
| --- | --- | --- | --- | --- | --- |
| n | 361 | 175 | 108 | 56 | 17 |
| ISC total score^1^ | 63 [45,88] (n = 239) | 73 [59,95] (n = 115) | 48 [30,68] (n = 69) | 46 [34,61] (n = 41) | 89 [80,96] (n = 13) |
| ISC clinician score^2^ | 21 [9,31] (n = 253) | 29 [20,39] (n = 127) | 10 [7,21] (n = 70) | 6 [5,11] (n = 41) | 23 [18,28] (n = 14) |
| ISC patient score^3^ | 42 [26,56] (n = 338) | 46 [36,56] (n = 158) | 27 [11,52] (n = 106) | 36 [24,52] (n = 54) | 65 [60,74] (n = 15) |
| BEBS total score^4^ | 24 [12,37] (n = 328) | 36 [27,44] (n = 154) | 13 [6,23] (n = 101) | 12 [8,16] (n = 53) | 27 [23,36] (n = 15) |
| ISC skin score^5^ | 7 [2,15] (n = 247) | 13 [9,20] (n = 115) | 2 [1,5] (n = 77) | 2 [0,4] (n = 41) | 11 [6,19] (n = 11) |
| BEBS skin score^6^ | 8 [2,14] (n = 329) | 14 [9,20] (n = 155) | 2 [0,6] (n = 101) | 1 [0,3] (n = 53) | 22 [14,26] (n = 15) |
| QOLEB total score^7^(adults only) | 18 [13,25] (n=240) | 24 [19,31] (n=81) | 14 [6,21] (n=91) | 15 [11,20] (n=49) | 30 [26,35] (n=14) |
| PedsQL total, ^8^ parent score | 48 [38,56] (n=86) | 47 [38,55] (n=81) | 57 [57,67] (n=5) |  |  |
| PedsQL total, ^8^ patient score | 54 [46,64] (n=63) | 54 [46,64] (n=61) | 57 [56,58] (n=2) |  |  |
| Annual dressing time, hrs | 364 [87,637] (n = 313) | 546 [364,910] (n = 173) | 61  [18,182] (n = 89) | 61  [12,121] (n = 29) | 520 [364,1274] (n = 17) |
| *Dressing frequency^9^* |  |  |  |  |  |
| - All at once | 270 (75) | 140 (80) | 80 (75) | 28 (51) | 17 (100) |
| - Few at a time | 46 (13) | 34 (20) | 9 (8) | 3 (5) | 0 (0) |
| - None required | 27 (8) | 0 (0) | 8 (7) | 19 (35) | 0 (0) |
| - Infrequent | 15 (4) | 0 (0) | 10 (9) | 5 (9) | 0 (0) |

*Results presented as n(%) or* *median [IQR] with participant numbers reported where results related to only some of the group*

*^1^ Total of iscorEB clinician and patient scores, maximum of 258*

*^2^ iscorEB clinician score, maximum of 138*

*^3^ iscorEB patient score, maximum of 120*

*^4^ BEBS, Birmingham EB Severity score, maximum of 100*

*^5^ Component of iscorEB clinician score, maximum of 78*

*^6^ Component of BEBS, maximum of 50*

*^7^ QOLEB, Quality of Life in Epidermolysis Bullosa questionnaire, maximum of 51*

*^8^ PedsQL, Pediatric Quality of Life Inventory, maximum of 100; higher score = lesser severity*

*^9^ Three missing scores*
