# Supplementary material for: Pain in recessive dystrophic epidermolysis bullosa (RDEB): findings of the Prospective Epidermolysis Bullosa Longitudinal Evaluation Study (PEBLES)
Source: Orphanet J Rare Dis. 2024 Oct 11;19:375. doi: 10.1186/s13023-024-03349-w (PMC11468479; doi:10.1186/s13023-024-03349-w)
Supplement: Supplementary file 2 — Supplementary Material 2 [file 13023_2024_3349_MOESM2_ESM.docx]

**Supplementary Table 2. Background and procedural pain VAS by RDEB subtype considering all reviews (n=361).**

| Variable | Category | Overall | RDEB-S | RDEB-I | RDEB-Inv | RDEB-Pru |
| --- | --- | --- | --- | --- | --- | --- |
| n |  | 361 | 175 | 108 | 56 | 17 |
| Background and/or procedural pain (VAS>0mm)^1^ | | 309 (93) | 158 (98) | 82 (81) | 49 (96) | 15 (100) |
| Background pain VAS^2^ | | 40 [20,60] (n = 335) | 40 [25,60] (n = 162) | 30 [0,60] (n=101) | 40 [20,60] (n = 52) | 55 [48,72] (n = 15) |
| Procedural pain VAS | | 60 [35,80] (n = 297) | 65 [40,80] (n = 164) | 40 [20,62] (n = 83) | 58 [30,69] (n = 30) | 85 [80,95] (n = 15) |
| VAS difference between procedural^3^ and background pain (Procedural – Background pain) | | 10 [0,25] (n = 294) | 20 [5,30] (n = 161) | 0 [0,20] (n = 83) | 5 [0,16] (n = 30) | 25 [10,42] (n=15) |

*VAS, visual analogue scale measured from 0-100mm.*

*Results are presented as n (%) or median [IQR] (n), reporting data used in figure 1.*

*Participant numbers reported where results related to only some of the group.*

*^1^ Another 25 reviews from 11 participants reported no background or procedural pain VAS, and 27 reviews were missing both pain VAS*

*^2^ All participants, regardless of dressing change frequency.*

*^3^ Excludes participants reporting no/infrequent dressing change*
