# Supplementary material for: Pain in recessive dystrophic epidermolysis bullosa (RDEB): findings of the Prospective Epidermolysis Bullosa Longitudinal Evaluation Study (PEBLES)
Source: Orphanet J Rare Dis. 2024 Oct 11;19:375. doi: 10.1186/s13023-024-03349-w (PMC11468479; doi:10.1186/s13023-024-03349-w)
Supplement: Supplementary file 3 — Supplementary Material 3 [file 13023_2024_3349_MOESM3_ESM.docx]

**Supplementary Table 3. Correlations between VAS background pain scores and severity scores by subtype when considering all reviews (n=361).**

| Variable 1 | Variable 2 | Overall | RDEB-S | RDEB-I | RDEB-Inv | RDEB-Pru |
| --- | --- | --- | --- | --- | --- | --- |
| Background pain VAS^1^ | iscorEB clinician score^3^ | *0.31 [0.19,0.42] (n = 234)* | *0.41 [0.25,0.55] (n = 120)* | *0.32 [0.08,0.52] (n = 64)* | *0.34 [0.02,0.60] (n = 37)* | 0.40 [-0.23,0.79] (n = 12) |
| ISP overall pain^2^ | iscorEB clinician score^3^ | *0.32 [0.19,0.45] (n = 176)* | *0.39 [0.20,0.56] (n = 86)* | *0.37 [0.09,0.59] (n = 48)* | 0.12 [-0.25,0.45] (n = 31) | 0.12 [-0.55,0.70] (n = 10) |
| Background pain VAS^1^ | iscorEB patient score^4^ | **0.68 [0.62,0.74] (n = 317)** | **0.51 [0.38,0.62] (n = 150)** | **0.77 [0.68,0.84] (n = 99)** | **0.80 [0.67,0.88] (n = 50)** | 0.20 [-0.39,0.68] (n = 13) |
| ISP overall pain^2^ | iscorEB patient score^4^ | **0.79 [0.74,0.83] (n = 259)** | **0.57 [0.43,0.68] (n = 121)** | **0.89 [0.83,0.93] (n = 78)** | **0.76 [0.60,0.86] (n = 44)** | **0.77 [0.35,0.93] (n = 12)** |
| Background pain VAS^1^ | iscorEB total score^5^ | **0.65 [0.56,0.72] (n = 223)** | **0.59 [0.46,0.70] (n = 111)** | **0.77 [0.65,0.86] (n = 63)** | **0.81 [0.67,0.90] (n = 37)** | 0.38 [-0.29,0.80] (n = 11) |
| ISP overall pain^2^ | iscorEB total score^5^ | **0.68 [0.59,0.75] (n = 171)** | **0.58 [0.42,0.71] (n = 82)** | **0.80 [0.66,0.88] (n = 47)** | **0.70 [0.47,0.85] (n = 31)** | 0.39 [-0.31,0.82] (n = 10) |
| Background pain VAS^1^ | iscorEB skin score^6^ | *0.39 [0.28,0.50] (n = 225)* | *0.39 [0.22,0.54] (n = 106)* | **0.55 [0.36,0.69] (n = 70)** | 0.13 [-0.21,0.43] (n = 37) | 0.18 [-0.55,0.76] (n = 9) |
| ISP overall pain^2^ | iscorEB skin score^6^ | *0.39 [0.27,0.49] (n = 236)* | *0.35 [0.17,0.51] (n = 109)* | *0.45 [0.24,0.61] (n = 74)* | 0.12 [-0.20,0.41] (n = 41) | 0.17 [-0.56,0.75] (n = 9) |
| Background pain VAS^1^ | BEBS total score^7^ | *0.38 [0.28,0.47] (n = 306)* | 0.25 [0.09,0.40] (n = 144) | **0.55 [0.40,0.68] (n = 95)** | *0.31 [0.03,0.55] (n = 49)* | 0.14 [-0.44,0.64] (n = 13) |
| ISP overall pain^2^ | BEBS total score^7^ | *0.42 [0.31,0.51] (n = 243)* | 0.28 [0.10,0.45] (n = 111) | **0.55 [0.36,0.69] (n = 75)** | 0.24 [-0.07,0.50] (n = 43) | 0.28 [-0.43,0.77] (n = 10) |
| Background pain VAS^1^ | BEBS skin score^8^ | *0.36 [0.26,0.46] (n = 307)* | 0.27 [0.11,0.41] (n = 145) | *0.49 [0.32,0.63] (n = 95)* | 0.12 [-0.17,0.39] (n = 49) | 0.17 [-0.42,0.66] (n = 13) |
| ISP overall pain^2^ | BEBS skin score^8^ | *0.40 [0.29,0.50] (n = 244)* | *0.38 [0.21,0.53] (n = 112)* | *0.39 [0.18,0.56] (n = 75)* | 0.00 [-0.30,0.30] (n = 43) | 0.18 [-0.51,0.73] (n = 10) |

*Variable 1: Patient-reported pain scores,*

*^1^ VAS, visual analogue scale*

*^2^ Question 1 of iscorEB patient questionnaire*

*Variable 2: Clinician and self-reported severity scores,*

*^3^ iscorEB clinician score*

*^4^ iscorEB patient score*

*^5^ Total of iscorEB clinician and patient scores*

*^6^ Component of iscorEB clinician score*

*^7^ BEBS, Birmingham EB Severity score*

*^8^ Component of BEBS*

*Results presented as correlation [95% CI] (n), calculated using Spearman’s rank correlation.*

*Results are significant if 95% CI does not include 0; correlations where n<10 should be considered with caution as associations could be spurious.*

*Significant associations:* ***large*** *(bold text), r=.50-1.0; medium (italics), r=.30-.49. Associations not highlighted in groups where n<10.*
