# Supplementary material for: Pain in recessive dystrophic epidermolysis bullosa (RDEB): findings of the Prospective Epidermolysis Bullosa Longitudinal Evaluation Study (PEBLES)
Source: Orphanet J Rare Dis. 2024 Oct 11;19:375. doi: 10.1186/s13023-024-03349-w (PMC11468479; doi:10.1186/s13023-024-03349-w)
Supplement: Supplementary file 4 — Supplementary Material 4 [file 13023_2024_3349_MOESM4_ESM.docx]

**Supplementary Table 4. Correlations between VAS procedural pain scores, for those reporting dressing changes, and severity scores by subtype at index review (n=61).**

| Variable 1 | Variable 2 | Overall | RDEB-S | RDEB-I | RDEB-Inv | RDEB-Pru |
| --- | --- | --- | --- | --- | --- | --- |
| Procedural pain VAS | iscorEB clinician score^1^ | *0.41 [0.15,0.62] (n = 50)* | 0.40 [-0.01,0.70] (n = 23) | 0.35 [-0.14,0.70] (n = 18) | 0.80 [-0.70,1.00] (n = 4) | 1.00 [1.00,1.00] (n = 4) |
| Procedural pain VAS | iscorEB patient score^2^ | **0.70 [0.53,0.81] (n = 54)** | **0.61 [0.28,0.81] (n = 25)** | **0.61 [0.23,0.83] (n = 20)** | 1.00 [1.00,1.00] (n = 4) | 0.80 [-0.70,1.00] (n = 4) |
| Procedural pain VAS | iscorEB total score^3^ | **0.66 [0.46,0.79] (n = 50)** | **0.64 [0.31,0.83] (n = 23)** | **0.53 [0.08,0.80] (n = 18)** | 1.00 [1.00,1.00] (n = 4) | 0.80 [-0.70,1.00] (n = 4) |
| Procedural pain VAS | iscorEB skin score^4^ | *0.46 [0.22,0.66] (n = 51)* | 0.39 [-0.02,0.69] (n = 23) | 0.40 [-0.07,0.72] (n = 19) | 0.40 [-0.91,0.98] (n = 4) | 1.00 [1.00,1.00] (n = 4) |
| Procedural pain VAS | BEBS total score^5^ | *0.39 [0.14,0.60] (n = 53)* | 0.36 [-0.06,0.66] (n = 24) | 0.41 [-0.04,0.72] (n = 20) | 0.80 [-0.70,1.00] (n = 4) | 1.00 [1.00,1.00] (n = 4) |
| Procedural pain VAS | BEBS skin score^6^ | *0.41 [0.16,0.61] (n = 53)* | 0.33 [-0.09,0.65] (n = 24) | 0.37 [-0.08,0.70] (n = 20) | 0.40 [-0.91,0.98] (n = 4) | 1.00 [1.00,1.00] (n = 4) |
| Procedural pain VAS | Dressing time (hrs) | *0.44 [0.20,0.64] (n = 53)* | 0.31 [-0.10,0.63] (n = 25) | 0.40 [-0.05,0.71] (n = 20) | n/a (n = 3) | 1.00 [1.00,1.00] (n = 4) |

*All reviews reporting frequent dressing changes were considered.*

*Variable 1: Patient-reported pain scores, VAS, visual analogue scale*

*Variable 2:* *Clinician and self-reported severity scores*

*^1^ iscorEB clinician score*

*^2^ iscorEB patient score*

*^3^ Total of iscorEB clinician and patient scores*

*^4^ Component of iscorEB clinician score*

*^5^ BEBS, Birmingham EB Severity score*

*^6^ Component of BEBS*

*Results presented as correlation [95% CI] (n), calculated using Spearman’s rank correlation.*

*Results are significant if 95% CI does not include 0; correlations where n<10 should be considered with caution as associations could be spurious.*

*Significant associations:* ***large*** *(bold text), r=.50-1.0; medium (italics), r=.30-.49. Associations not highlighted in groups where n<10.*
