# Supplementary material for: Pain in recessive dystrophic epidermolysis bullosa (RDEB): findings of the Prospective Epidermolysis Bullosa Longitudinal Evaluation Study (PEBLES)
Source: Orphanet J Rare Dis. 2024 Oct 11;19:375. doi: 10.1186/s13023-024-03349-w (PMC11468479; doi:10.1186/s13023-024-03349-w)
Supplement: Supplementary file 5 — Supplementary Material 5 [file 13023_2024_3349_MOESM5_ESM.docx]

**Supplementary Table 5. Correlations between VAS procedural pain scores and severity scores by subtype considering all reviews reporting regular dressing changes (n=316).**

| Variable 1 | Variable 2 | Overall | RDEB-S | RDEB-I | RDEB-Inv | RDEB-Pru |
| --- | --- | --- | --- | --- | --- | --- |
| Procedural pain VAS | iscorEB clinician score^1^ | *0.37 [0.25,0.48] (n = 215)* | *0.47 [0.32,0.59] (n = 123)* | 0.09 [-0.17,0.35] (n = 56) | **0.81 [0.59,0.92] (n = 23)** | -0.15 [-0.66,0.47] (n = 12) |
| Procedural pain VAS | iscorEB patient score^2^ | **0.67 [0.61,0.73] (n = 280)** | **0.54 [0.41,0.64] (n = 152)** | **0.74 [0.63,0.83] (n = 81)** | **0.80 [0.62,0.90] (n = 29)** | 0.44 [-0.15,0.80] (n = 13) |
| Procedural pain VAS | iscorEB total score^3^ | **0.69 [0.62,0.76] (n = 203)** | **0.65 [0.52,0.74] (n = 113)** | **0.64 [0.45,0.77] (n = 55)** | **0.85 [0.67,0.93] (n = 23)** | 0.34 [-0.32,0.78] (n = 11) |
| Procedural pain VAS | iscorEB skin score^4^ | *0.43 [0.31,0.54] (n = 198)* | *0.38 [0.21,0.53] (n = 108)* | *0.40 [0.15,0.60] (n = 57)* | **0.50 [0.09,0.77] (n = 21)** | 0.03 [-0.65,0.68] (n = 9) |
| Procedural pain VAS | BEBS total score^5^ | *0.45 [0.35,0.54] (n = 270)* | *0.33 [0.18,0.47] (n = 147)* | *0.46 [0.26,0.62] (n = 77)* | **0.72 [0.47,0.86] (n = 28)** | 0.18 [-0.41,0.66] (n = 13) |
| Procedural pain VAS | BEBS skin score^6^ | *0.46 [0.36,0.55] (n = 271)* | *0.32 [0.16,0.46] (n = 148)* | *0.40 [0.19,0.57] (n = 77)* | **0.55 [0.23,0.77] (n = 28)** | 0.19 [-0.41,0.67] (n = 13) |
| Procedural pain VAS | Dressing time (hrs) | *0.42 [0.32,0.51] (n = 295)* | 0.21 [0.06,0.35] (n = 164) | **0.61 [0.45,0.73] (n = 83)** | **0.61 [0.30,0.80] (n = 28)** | -0.11 [-0.59,0.43] (n = 15) |

** All reviews reporting frequent dressing changes were considered.*

*Variable 1: Patient-reported pain scores**, VAS, visual analogue scale*

*Variable 2:* *Clinician and self-reported severity scores*

*^1^ iscorEB clinician score*

*^2^ iscorEB patient score*

*^3^ Total of iscorEB clinician and patient scores*

*^4^ Component of iscorEB clinician score*

*^5^ BEBS, Birmingham EB Severity score*

*^6^ Component of BEBS*

*Results presented as correlation [95% CI] (n), calculated using Spearman’s rank correlation.*

*Results are significant if 95% CI does not include 0; correlations where n<10 should be considered with caution as associations could be spurious.*

*Significant associations:* ***large*** *(bold text), r=.50-1.0; medium (italics), r=.30-.49.*
