# Supplementary material for: Pain in recessive dystrophic epidermolysis bullosa (RDEB): findings of the Prospective Epidermolysis Bullosa Longitudinal Evaluation Study (PEBLES)
Source: Orphanet J Rare Dis. 2024 Oct 11;19:375. doi: 10.1186/s13023-024-03349-w (PMC11468479; doi:10.1186/s13023-024-03349-w)
Supplement: Supplementary file 6 — Supplementary Material 6 [file 13023_2024_3349_MOESM6_ESM.docx]

**Supplementary Table 6. Correlations between QOL and background pain VAS scores by subtype considering all reviews reporting regular dressing changes (n=316).**

| Variable 1 | Variable 2 | Overall | RDEB-S | RDEB-I | RDEB-Inv | RDEB-Pru |
| --- | --- | --- | --- | --- | --- | --- |
| QOLEB functioning score^1^ | VAS Background pain | **0.57 [0.48,0.65] (n = 237)** | **0.54 [0.36,0.68] (n = 80)** | **0.69 [0.56,0.78] (n = 91)** | **0.83 [0.71,0.90] (n = 48)** | -0.37 [-0.77,0.22] (n = 13) |
| QOLEB emotions score^2^ | VAS Background pain | *0.45 [0.34,0.54] (n = 240)* | *0.38 [0.17,0.55] (n = 80)* | **0.53 [0.36,0.66] (n = 92)** | **0.50 [0.26,0.69] (n = 50)** | -0.15 [-0.65,0.44] (n = 13) |
| QOLEB total score^3^ | VAS Background pain | **0.60 [0.52,0.68] (n = 236)** | **0.58 [0.41,0.71] (n = 80)** | **0.67 [0.54,0.77] (n = 90)** | **0.85 [0.75,0.92] (n = 48)** | -0.38 [-0.77,0.21] (n = 13) |
| PedsQL physical (parent)^4^ | VAS Background pain | -0.20 [-0.41,0.02] (n = 79) | -0.13 [-0.35,0.10] (n = 74) | -0.72 [-0.98,0.45] (n = 5) |  |  |
| PedsQL physical (patient)^4^ | VAS Background pain | -0.10 [-0.34,0.16] (n = 59) | -0.11 [-0.36,0.15] (n = 57) | n/a (n = 2) |  |  |
| PedsQL psychosocial (parent)^5^ | VAS Background pain | -0.21 [-0.41,0.01] (n = 78) | -0.15 [-0.37,0.08] (n = 73) | -0.72 [-0.98,0.45] (n = 5) |  |  |
| PedsQL psychosocial (patient)^5^ | VAS Background pain | -0.13 [-0.37,0.14] (n = 58) | -0.13 [-0.38,0.14] (n = 56) | n/a (n = 2) |  |  |
| PedsQL total score (parent)^6^ | VAS Background pain | -0.23 [-0.43,-0.01] (n = 78) | -0.17 [-0.38,0.06] (n = 73) | -0.72 [-0.98,0.45] (n = 5) |  |  |
| PedsQL total score (patient)^6^ | VAS Background pain | -0.14 [-0.38,0.12] (n = 58) | -0.16 [-0.40,0.11] (n = 56) | n/a (n = 2) |  |  |

*Variable 1: Quality of life self-report scores,*

*^1^ Subscore of QOLEB, Quality of Life in Epidermolysis Bullosa questionnaire*

*^2^ Subscore of QOLEB*

*^3^ Total of QOLEB*

*^4^ Physical health summary, a subscale of PedsQL, Pediatric Quality of Life Inventory*

*^5^ Psychosocial health summary, a subscale of PedsQL (comprising emotional, social and school functioning)*

*^6^ Total PedsQL score*

*Variable 2: Pain score, VAS, visual analogue scale*

*Results presented as correlation [95% CI] (n), calculated using Spearman’s rank correlation.*

*Results are significant if 95% CI does not include 0; correlations where n<10 should be considered with caution as associations could be spurious.*

*Significant associations:* ***large*** *(bold text), r=.50-1.0; medium (italics), r=.30-.49.*
