# Supplementary material for: Pain in recessive dystrophic epidermolysis bullosa (RDEB): findings of the Prospective Epidermolysis Bullosa Longitudinal Evaluation Study (PEBLES)
Source: Orphanet J Rare Dis. 2024 Oct 11;19:375. doi: 10.1186/s13023-024-03349-w (PMC11468479; doi:10.1186/s13023-024-03349-w)
Supplement: Supplementary file 7 — Supplementary Material 7 [file 13023_2024_3349_MOESM7_ESM.docx]

**Supplementary Table 7. Correlations between QOL and procedural pain VAS scores by subtype considering all reviews reporting regular dressing changes (n=316).**

| Variable 1 | Variable 2 | Overall | RDEB-S | RDEB-I | RDEB-Inv | RDEB-Pru |
| --- | --- | --- | --- | --- | --- | --- |
| QOLEB functioning score*^1^* | VAS Procedural pain | **0.67 [0.58,0.74] (n = 199)** | **0.63 [0.48,0.75] (n = 80)** | **0.64 [0.48,0.76] (n = 73)** | **0.79 [0.60,0.90] (n = 28)** | -0.19 [-0.67,0.40] (n = 13) |
| QOLEB emotions score*^2^* | VAS Procedural pain | *0.48 [0.36,0.58] (n = 201)* | *0.33 [0.12,0.51] (n = 80)* | *0.49 [0.29,0.64] (n = 74)* | **0.72 [0.49,0.86] (n = 29)** | 0.37 [-0.23,0.77] (n = 13) |
| QOLEB total score*^3^* | VAS Procedural pain | **0.71 [0.63,0.77] (n = 198)** | **0.63 [0.48,0.75] (n = 80)** | **0.63 [0.47,0.75] (n = 72)** | **0.81 [0.63,0.91] (n = 28)** | -0.03 [-0.57,0.53] (n = 13) |
| PedsQL physical (parent)*^4^* | VAS Procedural pain | -0.18 [-0.38,0.04] (n = 82) | -0.12 [-0.33,0.11] (n = 77) | -0.80 [-0.99,0.28] (n = 5) |  |  |
| PedsQL physical (patient)*^4^* | VAS Procedural pain | -0.11 [-0.36,0.14] (n = 61) | -0.12 [-0.36,0.15] (n = 59) | n/a (n = 2) |  |  |
| PedsQL psychosocial (parent)*^5^* | VAS Procedural pain | -0.18 [-0.39,0.04] (n = 81) | -0.13 [-0.35,0.09] (n = 76) | -0.80 [-0.99,0.28] (n = 5) |  |  |
| PedsQL psychosocial (patient)*^5^* | VAS Procedural pain | -0.12 [-0.36,0.14] (n = 60) | -0.12 [-0.37,0.14] (n = 58) | n/a (n = 2) |  |  |
| PedsQL total score (parent)*^6^* | VAS Procedural pain | -0.19 [-0.39,0.03] (n = 81) | -0.13 [-0.35,0.10] (n = 76) | -0.80 [-0.99,0.28] (n = 5) |  |  |
| PedsQL total score (patient)*^6^* | VAS Procedural pain | -0.15 [-0.39,0.11] (n = 60) | -0.15 [-0.39,0.11] (n = 58) | n/a (n = 2) |  |  |

*Variable 1: Quality of life self-report scores,*

*^1^ Subscore of QOLEB, Quality of Life in Epidermolysis Bullosa questionnaire*

*^2^ Subscore of QOLEB*

*^3^ Total of QOLEB*

*^4^ Physical health summary, a subscale of PedsQL, Pediatric Quality of Life Inventory*

*^5^ Psychosocial health summary, a subscale of PedsQL (comprising emotional, social and school functioning)*

*^6^ Total PedsQL score*

*Variable 2: Pain score, VAS, visual analogue scale; only participants with frequent dressing changes were included in procedural pain correlations.*

*Results presented as correlation [95% CI] (n), calculated using Spearman’s rank correlation. Results are significant if 95% CI does not include 0; correlations where n<10 should be considered with caution as associations could be spurious.*

*Significant associations:* ***large*** *(bold text), r=.50-1.0; medium (italics), r=.30-.49.*
