# Supplementary material for: Pain in recessive dystrophic epidermolysis bullosa (RDEB): findings of the Prospective Epidermolysis Bullosa Longitudinal Evaluation Study (PEBLES)
Source: Orphanet J Rare Dis. 2024 Oct 11;19:375. doi: 10.1186/s13023-024-03349-w (PMC11468479; doi:10.1186/s13023-024-03349-w)
Supplement: Supplementary file 8 — Supplementary Material 8 [file 13023_2024_3349_MOESM8_ESM.docx]

**Supplementary Table 8. Pain frequency by RDEB subtype for all reviews (n=361).**

| Variable | Category | Overall | RDEB-S | RDEB-I | RDEB-Inv | RDEB-Pru |
| --- | --- | --- | --- | --- | --- | --- |
| n |  | 361 | 175 | 108 | 56 | 17 |
| Weekly sleep disturbed pain,^1^ | 0 nights | 117 (34) | 40 (24) | 58 (56) | 18 (33) | 1 (6) |
|  | 1-3 nights | 100 (29) | 59 (36) | 21 (20) | 19 (35) | 1 (6) |
|  | 4-6 nights | 45 (13) | 26 (16) | 9 (9) | 5 (9) | 4 (25) |
|  | Every night | 82 (24) | 40 (24) | 16 (15) | 12 (22) | 10 (62) |
| Frequency of EB causing physical pain (QOLEB Q3)^2^ | No pain | 19 (8) | 0 (0) | 15 (16) | 3 (6) | 1 (7) |
|  | Occasional pain | 91 (37) | 30 (37) | 40 (43) | 20 (39) | 0 (0) |
|  | Frequent pain | 70 (29) | 28 (35) | 24 (26) | 14 (27) | 2 (14) |
|  | Constant pain | 65 (27) | 23 (28) | 15 (16) | 14 (27) | 11 (79) |
| Frequency of aches and pains? (PedsQL parent)^3^ | Never | 2 (2) | 1 (1) | 1 (20) |  |  |
|  | Almost never | 10 (11) | 10 (12) | 0 (0) |  |  |
|  | Sometimes | 33 (38) | 31 (38) | 2 (40) |  |  |
|  | Often | 25 (29) | 23 (28) | 2 (40) |  |  |
|  | Almost always | 17 (20) | 17 (21) | 0 (0) |  |  |
| Frequency of aches and pains? (PedsQL patient)^4^ | Never | 2 (3) | 2 (3) | 0 (0) |  |  |
|  | Almost never | 5 (8) | 5 (8) | 0 (0) |  |  |
|  | Sometimes | 29 (45) | 29 (47) | 0 (0) |  |  |
|  | Often | 12 (19) | 10 (16) | 2 (100) |  |  |
|  | Almost always | 16 (25) | 16 (26) | 0 (0) |  |  |

*Results are presented as n (%). All reviews are considered. Participant with RDEB-PT is only included in the ‘Overall’ subtype category.*

*^1^ n=344; missing data in 17 reviews*

*^2^ n=245; missing QOLEB (Quality of Life in Epidermolysis Bullosa questionnaire) data in 15 adult reviews*

*^3^ n=87; PedsQL (Pediatric Quality of Life Inventory) data not required for 8 child reviews (age<2y) and missing from 6 reviews*

*^4^ n=64; PedsQL data not required for 22 child reviews (age<4y) and missing from 15 reviews*
