# Supplementary material for: Pain in recessive dystrophic epidermolysis bullosa (RDEB): findings of the Prospective Epidermolysis Bullosa Longitudinal Evaluation Study (PEBLES)
Source: Orphanet J Rare Dis. 2024 Oct 11;19:375. doi: 10.1186/s13023-024-03349-w (PMC11468479; doi:10.1186/s13023-024-03349-w)
Supplement: Supplementary file 9 — Supplementary Material 9 [file 13023_2024_3349_MOESM9_ESM.docx]

**Supplementary Table 9. Pain location and intensity by RDEB subtype, data used for figure 2.**

| Variable^1^ | Category | Overall | RDEB-S | RDEB-I | RDEB-Inv | RDEB-Pru |
| --- | --- | --- | --- | --- | --- | --- |
| **Considering index reviews, n** | | **61** | **25** | **22** | **9** | **4** |
| Overall pain | None | 6 (10) | 0 (0) | 5 (24) | 0 (0) | 1 (25) |
|  | Mild | 17 (29) | 6 (25) | 7 (33) | 4 (44) | 0 (0) |
|  | Moderate | 13 (22) | 7 (29) | 4 (19) | 1 (11) | 1 (25) |
|  | Severe | 17 (29) | 10 (42) | 4 (19) | 3 (33) | 0 (0) |
|  | Worst possible | 6 (10) | 1 (4) | 1 (5) | 1 (11) | 2 (50) |
| Skin pain | None | 7 (12) | 0 (0) | 5 (24) | 1 (11) | 1 (25) |
|  | Mild | 14 (24) | 3 (12) | 7 (33) | 4 (44) | 0 (0) |
|  | Moderate | 16 (27) | 9 (38) | 5 (24) | 1 (11) | 1 (25) |
|  | Severe | 17 (29) | 10 (42) | 4 (19) | 3 (33) | 0 (0) |
|  | Worst possible | 5 (8) | 2 (8) | 0 (0) | 0 (0) | 2 (50) |
| Mouth pain | None | 9 (15) | 2 (8) | 5 (24) | 0 (0) | 1 (25) |
|  | Mild | 18 (31) | 9 (38) | 6 (29) | 3 (33) | 0 (0) |
|  | Moderate | 16 (27) | 6 (25) | 5 (24) | 3 (33) | 2 (50) |
|  | Severe | 12 (20) | 7 (29) | 3 (14) | 2 (22) | 0 (0) |
|  | Worst possible | 4 (7) | 0 (0) | 2 (10) | 1 (11) | 1 (25) |
| Eye pain | None | 33 (56) | 11 (46) | 14 (67) | 4 (44) | 3 (75) |
|  | Mild | 14 (24) | 8 (33) | 2 (10) | 4 (44) | 0 (0) |
|  | Moderate | 8 (14) | 3 (12) | 4 (19) | 1 (11) | 0 (0) |
|  | Severe | 4 (7) | 2 (8) | 1 (5) | 0 (0) | 1 (25) |
|  | Worst possible | 0 (0) | 0 (0) | 0 (0) | 0 (0) | 0 (0) |
| Bone/Joint pain | None | 27 (46) | 11 (46) | 9 (43) | 5 (56) | 2 (50) |
|  | Mild | 17 (29) | 8 (33) | 8 (38) | 1 (11) | 0 (0) |
|  | Moderate | 6 (10) | 1 (4) | 3 (14) | 1 (11) | 1 (25) |
|  | Severe | 8 (14) | 3 (12) | 1 (5) | 2 (22) | 1 (25) |
|  | Worst possible | 1 (2) | 1 (4) | 0 (0) | 0 (0) | 0 (0) |

| Variable^1^ | Category | Overall | RDEB-S | RDEB-I | RDEB-Inv | RDEB-Pru |
| --- | --- | --- | --- | --- | --- | --- |
| **Considering all reviews, n** | | **361** | **175** | **108** | **56** | **17** |
| Overall pain | None | 30 (11) | 5 (4) | 22 (28) | 2 (4) | 1 (8) |
|  | Mild | 65 (24) | 27 (21) | 25 (31) | 13 (29) | 0 (0) |
|  | Moderate | 83 (31) | 46 (36) | 16 (20) | 18 (40) | 1 (8) |
|  | Severe | 67 (25) | 43 (34) | 13 (16) | 7 (16) | 3 (25) |
|  | Worst possible | 23 (9) | 6 (5) | 4 (5) | 5 (11) | 7 (58) |
| Skin pain | None | 33 (12) | 5 (4) | 22 (28) | 5 (11) | 1 (8) |
|  | Mild | 59 (22) | 19 (15) | 25 (31) | 15 (33) | 0 (0) |
|  | Moderate | 86 (32) | 51 (40) | 17 (21) | 17 (38) | 1 (8) |
|  | Severe | 66 (25) | 44 (35) | 12 (15) | 3 (7) | 4 (33) |
|  | Worst possible | 23 (9) | 7 (6) | 4 (5) | 5 (11) | 6 (50) |
| Mouth pain | None | 53 (20) | 21 (17) | 22 (28) | 5 (11) | 1 (8) |
|  | Mild | 89 (33) | 53 (42) | 21 (26) | 9 (20) | 6 (50) |
|  | Moderate | 79 (29) | 37 (29) | 22 (28) | 17 (38) | 3 (25) |
|  | Severe | 35 (13) | 16 (13) | 9 (11) | 9 (20) | 1 (8) |
|  | Worst possible | 12 (4) | 0 (0) | 6 (8) | 5 (11) | 1 (8) |
| Eye pain | None | 136 (51) | 53 (42) | 52 (65) | 18 (40) | 9 (75) |
|  | Mild | 87 (32) | 50 (39) | 15 (19) | 20 (44) | 2 (17) |
|  | Moderate | 25 (9) | 13 (10) | 6 (8) | 6 (13) | 0 (0) |
|  | Severe | 17 (6) | 10 (8) | 5 (6) | 1 (2) | 1 (8) |
|  | Worst possible | 3 (1) | 1 (1) | 2 (2) | 0 (0) | 0 (0) |
| Bone/Joint pain | None | 120 (45) | 60 (47) | 36 (46) | 21 (47) | 3 (25) |
|  | Mild | 69 (26) | 36 (28) | 25 (32) | 6 (13) | 1 (8) |
|  | Moderate | 36 (13) | 17 (13) | 8 (10) | 8 (18) | 3 (25) |
|  | Severe | 36 (13) | 13 (10) | 7 (9) | 8 (18) | 5 (42) |
|  | Worst possible | 6 (2) | 1 (1) | 3 (4) | 2 (4) | 0 (0) |

*Results are presented as n (%). Index and all reviews are considered. Participant with RDEB-PT is only included in the ‘Overall’ subtype category.*

*^1^ These are the first 5 questions on the iscorEB patient questionnaire*
