# Supplementary material for: Pain in recessive dystrophic epidermolysis bullosa (RDEB): findings of the Prospective Epidermolysis Bullosa Longitudinal Evaluation Study (PEBLES)
Source: Orphanet J Rare Dis. 2024 Oct 11;19:375. doi: 10.1186/s13023-024-03349-w (PMC11468479; doi:10.1186/s13023-024-03349-w)
Supplement: Supplementary file 10 — Supplementary Material 10 [file 13023_2024_3349_MOESM10_ESM.docx]

**Supplementary Table 10. Correlations between pain location and severity scores by subtype at index review (n=61).**

| Variable 1^1^ | Variable 2 | Overall | RDEB-S | RDEB-I | RDEB-Inv | RDEB-Pru |
| --- | --- | --- | --- | --- | --- | --- |
| Skin pain | BEBS total score^2^ | **0.52 [0.30,0.68] (n = 58)** | 0.23 [-0.20,0.59] (n = 23) | **0.66 [0.32,0.85] (n = 21)** | 0.32 [-0.43,0.81] (n = 9) | 0.95 [-0.14,1.00] (n = 4) |
| Mouth pain | BEBS total score^2^ | 0.12 [-0.14,0.37] (n = 58) | -0.05 [-0.45,0.37] (n = 23) | 0.36 [-0.08,0.69] (n = 21) | 0.36 [-0.40,0.83] (n = 9) | 0.40 [-0.91,0.98] (n = 4) |
| Eye pain | BEBS total score^2^ | 0.16 [-0.11,0.40] (n = 58) | -0.05 [-0.45,0.37] (n = 23) | 0.26 [-0.19,0.62] (n = 21) | 0.47 [-0.28,0.86] (n = 9) | 0.26 [-0.93,0.98] (n = 4) |
| Bone/Joint pain | BEBS total score^2^ | 0.19 [-0.08,0.42] (n = 58) | 0.18 [-0.25,0.55] (n = 23) | *0.48 [0.06,0.75] (n = 21)* | 0.10 [-0.60,0.72] (n = 9) | 0.11 [-0.95,0.97] (n = 4) |
| Skin pain | BEBS skin score^3^ | **0.55 [0.34,0.71] (n = 58)** | 0.23 [-0.20,0.59] (n = 23) | **0.66 [0.32,0.85] (n = 21)** | 0.34 [-0.42,0.82] (n = 9) | 0.95 [-0.14,1.00] (n = 4) |
| Mouth pain | BEBS skin score^3^ | 0.05 [-0.21,0.30] (n = 58) | -0.02 [-0.43,0.40] (n = 23) | 0.23 [-0.22,0.60] (n = 21) | -0.13 [-0.73,0.58] (n = 9) | 0.40 [-0.91,0.98] (n = 4) |
| Eye pain | BEBS skin score^3^ | 0.08 [-0.19,0.33] (n = 58) | -0.10 [-0.49,0.33] (n = 23) | 0.11 [-0.34,0.52] (n = 21) | 0.37 [-0.39,0.83] (n = 9) | 0.26 [-0.93,0.98] (n = 4) |
| Bone/Joint pain | BEBS skin score^3^ | 0.17 [-0.09,0.41] (n = 58) | 0.17 [-0.26,0.54] (n = 23) | *0.44 [0.01,0.73] (n = 21)* | 0.02 [-0.65,0.67] (n = 9) | 0.11 [-0.95,0.97] (n = 4) |
| Skin pain | Dressing time (hrs) | **0.60 [0.39,0.75] (n = 51)** | 0.37 [-0.04,0.67] (n = 24) | **0.58 [0.17,0.82] (n = 19)** | n/a (n = 3) | 0.95 [-0.14,1.00] (n = 4) |
| Mouth pain | Dressing time (hrs) | 0.26 [-0.02,0.50] (n = 51) | 0.40 [-0.01,0.69] (n = 24) | 0.08 [-0.39,0.51] (n = 19) | n/a (n = 3) | 0.40 [-0.91,0.98] (n = 4) |
| Eye pain | Dressing time (hrs) | 0.13 [-0.15,0.39] (n = 51) | 0.08 [-0.33,0.47] (n = 24) | -0.10 [-0.53,0.37] (n = 19) | n/a (n = 3) | 0.26 [-0.93,0.98] (n = 4) |
| Bone/Joint pain | Dressing time (hrs) | 0.19 [-0.09,0.44] (n = 51) | *0.44 [0.04,0.72] (n = 24)* | 0.15 [-0.33,0.56] (n = 19) | n/a (n = 3) | 0.11 [-0.95,0.97] (n = 4) |

*^1^ These are questions 2-5 on the iscorEB patient questionnaire*

*^2^ BEBS, Birmingham EB Severity score*

*^3^ Component of BEBS*

*Results presented as correlation [95% CI] (n), calculated using Spearman’s rank correlation.*

*Results are significant if 95% CI does not include 0; correlations where n<10 should be considered with caution as associations could be spurious.*

*Significant associations:* **large** *(bold text), r=.50-1.0; medium (italics), r=.30-.49.*
