# Supplementary material for: Pain in recessive dystrophic epidermolysis bullosa (RDEB): findings of the Prospective Epidermolysis Bullosa Longitudinal Evaluation Study (PEBLES)
Source: Orphanet J Rare Dis. 2024 Oct 11;19:375. doi: 10.1186/s13023-024-03349-w (PMC11468479; doi:10.1186/s13023-024-03349-w)
Supplement: Supplementary file 11 — Supplementary Material 11 [file 13023_2024_3349_MOESM11_ESM.docx]

**Supplementary Table 11. Correlations between pain location and severity scores by subtype when considering all reviews (n=361).**

| Variable 1^1^ | Variable 2 | Overall | RDEB-S | RDEB-I | RDEB-Inv | RDEB-Pru |
| --- | --- | --- | --- | --- | --- | --- |
| Skin pain | BEBS total score^2^ | *0.47 [0.36,0.56] (n = 242)* | 0.22 [0.04,0.39] (n = 110) | 0.55 [0.36,0.69] (n = 75) | *0.36 [0.07,0.60] (n = 43)* | 0.29 [-0.41,0.78] (n = 10) |
| Mouth pain | BEBS total score^2^ | 0.09 [-0.03,0.22] (n = 243) | 0.06 [-0.13,0.24] (n = 111) | *0.41 [0.21,0.59] (n = 75)* | *0.38 [0.09,0.61] (n = 43)* | -0.03 [-0.65,0.61] (n = 10) |
| Eye pain | BEBS total score^2^ | 0.20 [0.07,0.32] (n = 243) | 0.19 [-0.00,0.36] (n = 111) | 0.18 [-0.05,0.39] (n = 75) | *0.31 [0.02,0.56] (n = 43)* | -0.35 [-0.80,0.36] (n = 10) |
| Bone/Joint pain | BEBS total score^2^ | 0.16 [0.03,0.28] (n = 242) | *0.30 [0.12,0.46] (n = 111)* | *0.43 [0.22,0.60] (n = 74)* | 0.08 [-0.22,0.37] (n = 43) | -0.28 [-0.77,0.43] (n = 10) |
| Skin pain | BEBS skin score^3^ | *0.48 [0.37,0.57] (n = 243)* | *0.30 [0.12,0.46] (n = 111)* | *0.42 [0.21,0.59] (n = 75)* | *0.39 [0.10,0.62] (n = 43)* | 0.26 [-0.44,0.76] (n = 10) |
| Mouth pain | BEBS skin score^3^ | -0.01 [-0.14,0.11] (n = 244) | 0.08 [-0.11,0.26] (n = 112) | 0.23 [0.01,0.44] (n = 75) | -0.01 [-0.31,0.29] (n = 43) | -0.13 [-0.70,0.54] (n = 10) |
| Eye pain | BEBS skin score^3^ | 0.11 [-0.01,0.24] (n = 244) | 0.23 [0.05,0.40] (n = 112) | -0.03 [-0.25,0.20] (n = 75) | 0.08 [-0.22,0.38] (n = 43) | -0.25 [-0.76,0.45] (n = 10) |
| Bone/Joint pain | BEBS skin score^3^ | 0.14 [0.02,0.27] (n = 243) | *0.34 [0.17,0.50] (n = 112)* | 0.27 [0.05,0.47] (n = 74) | 0.08 [-0.22,0.37] (n = 43) | -0.25 [-0.76,0.45] (n = 10) |
| Skin pain | Dressing time (hrs) | *0.31 [0.18,0.42] (n = 229)* | 0.02 [-0.16,0.19] (n = 126) | **0.51 [0.30,0.67] (n = 66)** | **0.54 [0.14,0.79] (n = 21)** | 0.18 [-0.44,0.68] (n = 12) |
| Mouth pain | Dressing time (hrs) | 0.12 [-0.01,0.24] (n = 230) | 0.20 [0.03,0.37] (n = 127) | *0.31 [0.07,0.51] (n = 66)* | **0.55 [0.15,0.79] (n = 21)** | 0.34 [-0.29,0.76] (n = 12) |
| Eye pain | Dressing time (hrs) | 0.13 [0.00,0.26] (n = 230) | 0.09 [-0.08,0.26] (n = 127) | 0.10 [-0.15,0.33] (n = 66) | -0.04 [-0.46,0.40] (n = 21) | -0.30 [-0.75,0.33] (n = 12) |
| Bone/Joint pain | Dressing time (hrs) | 0.05 [-0.08,0.17] (n = 229) | 0.12 [-0.05,0.29] (n = 127) | 0.27 [0.03,0.48] (n = 65) | 0.33 [-0.12,0.67] (n = 21) | -0.28 [-0.73,0.35] (n = 12) |

*^1^ These are questions 2-5 on the iscorEB patient questionnaire*

*^2^ BEBS, Birmingham EB Severity score*

*^3^ Component of BEBS*

*Results presented as correlation [95% CI] (n), calculated using Spearman’s rank correlation.*

*Results are significant if 95% CI does not include 0; correlations where n<10 should be considered with caution as associations could be spurious.*

*Significant associations:* **large** *(bold text), r=.50-1.0; medium (italics), r=.30-.49.*
