# Supplementary material for: Pain in recessive dystrophic epidermolysis bullosa (RDEB): findings of the Prospective Epidermolysis Bullosa Longitudinal Evaluation Study (PEBLES)
Source: Orphanet J Rare Dis. 2024 Oct 11;19:375. doi: 10.1186/s13023-024-03349-w (PMC11468479; doi:10.1186/s13023-024-03349-w)
Supplement: Supplementary file 12 — Supplementary Material 12 [file 13023_2024_3349_MOESM12_ESM.docx]

**Supplementary Table 12. Reported background and procedural pain VAS reported in Figure 3 according to medication usage at index review (n=61) and when considering all reviews (n=361).**

| ***Index review*** | | Overall | RDEB-S | RDEB-I | RDEB-Inv | RDEB-Pru |
| --- | --- | --- | --- | --- | --- | --- |
| **Pain medication** | **Background pain** | *(n=61)* | *(n=25)* | *(n=22)* | *(n=9)* | *(n=4)* |
| Strong opioids | No | 30 [20,60] (n = 43) | 33 [22,55] (n = 14) | 32 [8,61] (n = 20) | 30 [30,35] (n = 7) | 0 [0,0] (n = 1) |
|  | Yes | 50 [32,69] (n = 18) | 48 [25,50] (n = 11) | 85 [82,88] (n = 2) | 54 [42,65] (n = 2) | 60 [58,65] (n = 3) |
| Weak opioids | No | 38 [20,60] (n = 58) | 39 [20,54] (n = 25) | 40 [10,70] (n = 21) | 30 [30,32] (n = 8) | 58 [41,62] (n = 4) |
|  | Yes | 80 [60,80] (n = 3) |  | 40 [40,40] (n = 1) | 80 [80,80] (n = 1) |  |
| Non-opioid NSAID | No | 30 [20,56] (n = 44) | 49 [20,54] (n = 16) | 20 [0,50] (n = 17) | 30 [30,54] (n = 7) | 55 [28,58] (n = 3) |
|  | Yes | 50 [30,70] (n = 17) | 36 [30,50] (n = 9) | 70 [65,80] (n = 5) | 35 [32,38] (n = 2) | 70 [70,70] (n = 1) |
| Non-opioid paracetamol | No | 30 [20,54] (n = 30) | 39 [20,50] (n = 10) | 22 [15,48] (n = 12) | 30 [30,38] (n = 6) | 58 [56,59] (n = 2) |
|  | Yes | 50 [30,68] (n = 31) | 39 [30,57] (n = 15) | 60 [20,69] (n = 10) | 30 [30,54] (n = 3) | 35 [18,52] (n = 2) |
| Adjunctive | No | 30 [20,60] (n = 50) | 36 [20,52] (n = 19) | 40 [12,69] (n = 22) | 30 [30,30] (n = 7) | 30 [15,45] (n = 2) |
|  | Yes | 54 [44,70] (n = 11) | 49 [34,53] (n = 6) |  | 58 [49,68] (n = 2) | 62 [59,66] (n = 2) |

| ***Index review*** |  | Overall | RDEB-S | RDEB-I | RDEB-Inv | RDEB-Pru |
| --- | --- | --- | --- | --- | --- | --- |
| **Pain medication** | **Procedural pain** | *(n=61)* | *(n=25)* | *(n=22)* | *(n=9)* | *(n=4)* |
| Strong opioids | No | 50 [38,70] (n = 36) | 55 [40,69] (n = 14) | 45 [25,66] (n = 18) | 25 [12,38] (n = 2) | 10 [10,10] (n = 1) |
|  | Yes | 70 [50,84] (n = 18) | 70 [50,80] (n = 11) | 62 [51,74] (n = 2) | 48 [39,56] (n = 2) | 85 [82,88] (n = 3) |
| Weak opioids | No | 52 [40,80] (n = 52) | 60 [40,75] (n = 25) | 50 [30,75] (n = 19) | 40 [22,54] (n = 4) | 82 [62,86] (n = 4) |
|  | Yes | 60 [50,70] (n = 2) |  | 40 [40,40] (n = 1) |  |  |
| Non-opioid NSAID | No | 30 [20,56] (n = 44) | 49 [20,54] (n = 16) | 20 [0,50] (n = 17) | 30 [30,54] (n = 7) | 55 [28,58] (n = 3) |
|  | Yes | 50 [30,70] (n = 17) | 36 [30,50] (n = 9) | 70 [65,80] (n = 5) | 35 [32,38] (n = 2) | 70 [70,70] (n = 1) |
| Non-opioid paracetamol | No | 45 [38,80] (n = 24) | 45 [40,76] (n = 10) | 40 [25,54] (n = 10) | 25 [12,38] (n = 2) | 82 [81,84] (n = 2) |
|  | Yes | 62 [42,79] (n = 30) | 64 [50,72] (n = 15) | 52 [40,78] (n = 10) | 48 [39,56] (n = 2) | 50 [30,70] (n = 2) |
| Adjunctive | No | 50 [40,70] (n = 43) | 60 [45,70] (n = 19) | 45 [35,72] (n = 20) | 15 [8,22] (n = 2) | 48 [29,66] (n = 2) |
|  | Yes | 75 [58,80] (n = 11) | 72 [40,79] (n = 6) |  | 58 [54,61] (n = 2) | 85 [82,88] (n = 2) |

| ***All reviews*** |  | Overall | RDEB-S | RDEB-I | RDEB-Inv | RDEB-Pru |
| --- | --- | --- | --- | --- | --- | --- |
| **Pain medication** | **Background pain** | *(n=361)^1^* | *(n=175)* | *(n=108)* | *(n=56)* | *(n=17)* |
| Strong opioids | No | 30 [10,60] (n = 229) | 35 [20,60] (n = 90) | 20 [0,50] (n = 92) | 30 [20,52] (n = 40) | 50 [25,52] (n = 3) |
|  | Yes | 50 [36,70] (n = 106) | 42 [30,60] (n = 72) | 70 [60,80] (n = 9) | 60 [44,78] (n = 12) | 58 [49,79] (n = 12) |
| Weak opioids | No | 40 [20,60] (n = 324) | 40 [25,60] (n = 158) | 28 [0,60] (n = 98) | 40 [20,60] (n = 49) | 55 [48,72] (n = 15) |
|  | Yes | 60 [36,80] (n = 11) | 65 [50,78] (n = 4) | 32 [24,36] (n = 3) | 80 [70,82] (n = 3) |  |
| Non-opioid NSAID | No | 38 [20,60] (n = 246) | 40 [20,60] (n = 104) | 20 [0,40] (n = 81) | 40 [20,60] (n = 44) | 55 [49,79] (n = 12) |
|  | Yes | 50 [30,70] (n = 89) | 48 [30,69] (n = 58) | 60 [48,70] (n = 20) | 35 [20,45] (n = 8) | 50 [38,60] (n = 3) |
| Non-opioid paracetamol | No | 30 [10,60] (n = 175) | 40 [20,60] (n = 66) | 12 [0,40] (n = 62) | 30 [10,50] (n = 33) | 55 [51,71] (n = 10) |
|  | Yes | 50 [30,65] (n = 160) | 40 [29,60] (n = 96) | 50 [30,70] (n = 39) | 50 [40,64] (n = 19) | 50 [25,70] (n = 5) |
| Adjunctive | No | 34 [16,60] (n = 260) | 40 [20,60] (n = 113) | 25 [0,50] (n = 99) | 30 [20,50] (n = 39) | 55 [49,69] (n = 8) |
|  | Yes | 50 [38,70] (n = 75) | 50 [30,60] (n = 49) | 68 [64,71] (n = 2) | 69 [45,80] (n = 13) | 55 [38,72] (n = 7) |

| ***All reviews*** |  | Overall | RDEB-S | RDEB-I | RDEB-Inv | RDEB-Pru |
| --- | --- | --- | --- | --- | --- | --- |
| **Pain medication** | **Procedural pain** | *(n=319)^1^* | *(n=175)* | *(n=90)* | *(n=32)* | *(n=17)* |
| Strong opioids | No | 50 [30,70] (n = 190) | 60 [38,75] (n = 91) | 40 [20,60] (n = 74) | 40 [10,58] (n = 18) | 80 [45,90] (n = 3) |
|  | Yes | 70 [50,82] (n = 107) | 70 [50,80] (n = 73) | 80 [40,85] (n = 9) | 75 [64,86] (n = 12) | 85 [84,95] (n = 12) |
| Weak opioids | No | 60 [30,80] (n = 289) | 65 [40,80] (n = 160) | 40 [20,62] (n = 80) | 58 [30,69] (n = 30) | 85 [80,95] (n = 15) |
|  | Yes | 60 [50,80] (n = 8) | 65 [50,85] (n = 4) | 55 [48,60] (n = 3) |  |  |
| Non-opioid NSAID | No | 50 [30,75] (n = 210) | 64 [40,80] (n = 104) | 30 [15,52] (n = 63) | 58 [30,74] (n = 26) | 85 [80,96] (n = 12) |
|  | Yes | 65 [50,80] (n = 87) | 65 [50,80] (n = 60) | 65 [48,85] (n = 20) | 55 [48,61] (n = 4) | 85 [82,88] (n = 3) |
| Non-opioid paracetamol | No | 50 [30,80] (n = 139) | 64 [40,80] (n = 65) | 30 [2,50] (n = 46) | 40 [10,71] (n = 14) | 90 [85,99] (n = 10) |
|  | Yes | 60 [40,80] (n = 158) | 65 [50,80] (n = 99) | 50 [30,80] (n = 37) | 60 [40,66] (n = 16) | 80 [25,85] (n = 5) |
| Adjunctive | No | 50 [30,75] (n = 224) | 60 [40,80] (n = 115) | 40 [20,60] (n = 81) | 40 [10,60] (n = 19) | 95 [84,100] (n = 8) |
|  | Yes | 70 [60,80] (n = 73) | 70 [50,80] (n = 49) | 85 [80,90] (n = 2) | 65 [50,82] (n = 11) | 85 [80,85] (n = 7) |

*VAS, visual analogue scale measured from 0-100mm.*

*Results are presented as median [IQR]. Index and all reviews are considered.*

*Participant with RDEB-PT is only included in the ‘Overall’ subtype category.*
