# Supplementary material for: Pain in recessive dystrophic epidermolysis bullosa (RDEB): findings of the Prospective Epidermolysis Bullosa Longitudinal Evaluation Study (PEBLES)
Source: Orphanet J Rare Dis. 2024 Oct 11;19:375. doi: 10.1186/s13023-024-03349-w (PMC11468479; doi:10.1186/s13023-024-03349-w)
Supplement: Supplementary file 13 — Supplementary Material 13 [file 13023_2024_3349_MOESM13_ESM.docx]

**Supplementary Table 13. Reported background and procedural pain VAS according to regular or PRN medication usage at index review (n=61) and when considering all reviews (n=361).**

| Pain medication | Pain | Overall | RDEB-S | RDEB-I | RDEB-Inv | RDEB-Pru |
| --- | --- | --- | --- | --- | --- | --- |
| ***Index review*** | *Background* | *(n=61)* | *(n=25)* | *(n=22)* | *(n=9)* | *(n=4)* |
| All medications | None | 20 [15,35] (n = 19) | 20 [20,52] (n = 7) | 20 [0,25] (n = 9) | 30 [15,30] (n = 3) |  |
|  | Regular +/- PRN | 50 [38,70] (n = 19) | 44 [30,54] (n = 12) | 90 [90,90] (n = 1) | 58 [49,68] (n = 2) | 60 [58,65] (n = 3) |
|  | PRN only | 50 [30,62] (n = 23) | 40 [30,53] (n = 6) | 60 [32,70] (n = 12) | 30 [30,42] (n = 4) | 0 [0,0] (n = 1) |
| Strong opioids | None | 30 [20,60] (n = 43) | 33 [22,55] (n = 14) | 32 [8,61] (n = 20) | 30 [30,35] (n = 7) | 0 [0,0] (n = 1) |
|  | Regular +/- PRN | 60 [50,74] (n = 11) | 50 [35,61] (n = 6) | 90 [90,90] (n = 1) | 77 [77,77] (n = 1) | 60 [58,65] (n = 3) |
|  | PRN only | 39 [25,49] (n = 7) | 39 [20,48] (n = 5) | 80 [80,80] (n = 1) | 30 [30,30] (n = 1) |  |
| Weak opioids | None | 38 [20,60] (n = 58) | 39 [20,54] (n = 25) | 40 [10,70] (n = 21) | 30 [30,32] (n = 8) | 58 [41,62] (n = 4) |
|  | Regular/PRN | n/a |  |  |  |  |
|  | PRN only | 80 [60,80] (n = 3) |  | 40 [40,40] (n = 1) | 80 [80,80] (n = 1) |  |
| Non-opioid NSAID | None | 30 [20,56] (n = 44) | 49 [20,54] (n = 16) | 20 [0,50] (n = 17) | 30 [30,54] (n = 7) | 55 [28,58] (n = 3) |
|  | Regular +/- PRN | 50 [38,75] (n = 7) | 43 [34,58] (n = 4) | 90 [90,90] (n = 1) | 40 [40,40] (n = 1) | 70 [70,70] (n = 1) |
|  | PRN only | 50 [30,65] (n = 10) | 30 [20,39] (n = 5) | 68 [64,72] (n = 4) | 30 [30,30] (n = 1) |  |
| Non-opioid paracetamol | None | 30 [20,54] (n = 30) | 39 [20,50] (n = 10) | 22 [15,48] (n = 12) | 30 [30,38] (n = 6) | 58 [56,59] (n = 2) |
|  | Regular +/- PRN | 50 [34,74] (n = 11) | 44 [28,54] (n = 8) | 90 [90,90] (n = 1) | 77 [77,77] (n = 1) | 70 [70,70] (n = 1) |
|  | PRN only | 43 [25,61] (n = 20) | 36 [30,57] (n = 7) | 60 [10,65] (n = 9) | 30 [30,30] (n = 2) | 0 [0,0] (n = 1) |
| Adjunctive | None | 30 [20,60] (n = 50) | 36 [20,52] (n = 19) | 40 [12,69] (n = 22) | 30 [30,30] (n = 7) | 30 [15,45] (n = 2) |
|  | Regular +/- PRN | 55 [48,70] (n = 9) | 49 [44,55] (n = 4) |  | 58 [49,68] (n = 2) | 62 [59,66] (n = 2) |
|  | PRN only | 37 [28,46] (n = 2) | 37 [28,46] (n = 2) |  |  |  |

| Pain medication | Pain | Overall | RDEB-S | RDEB-I | RDEB-Inv | RDEB-Pru |
| --- | --- | --- | --- | --- | --- | --- |
| ***All reviews*** | *Background* | *(n=361)^1^* | *(n=175)* | *(n=108)* | *(n=56)* | *(n=17)* |
| All medications | None | 20 [0,40] (n = 106) | 35 [20,60] (n = 36) | 0 [0,24] (n = 50) | 20 [5,30] (n = 17) | 52 [51,54] (n = 2) |
|  | Regular +/- PRN | 55 [40,70] (n = 104) | 50 [30,61] (n = 64) | 70 [60,72] (n = 11) | 62 [46,79] (n = 14) | 60 [52,82] (n = 11) |
|  | PRN only | 40 [20,60] (n = 125) | 38 [20,55] (n = 62) | 40 [19,66] (n = 40) | 40 [30,60] (n = 21) | 12 [6,19] (n = 2) |
| Strong opioids | None | 30 [10,60] (n = 229) | 35 [20,60] (n = 90) | 20 [0,50] (n = 92) | 30 [20,52] (n = 40) | 50 [25,52] (n = 3) |
|  | Regular +/- PRN | 60 [46,75] (n = 54) | 50 [38,68] (n = 31) | 70 [60,80] (n = 5) | 77 [58,80] (n = 7) | 60 [52,82] (n = 11) |
|  | PRN only | 40 [30,60] (n = 52) | 40 [30,50] (n = 41) | 70 [52,72] (n = 4) | 40 [40,60] (n = 5) | 25 [25,25] (n = 1) |
| Weak opioids | None | 40 [20,60] (n = 324) | 40 [25,60] (n = 158) | 28 [0,60] (n = 98) | 40 [20,60] (n = 49) | 55 [48,72] (n = 15) |
|  | Regular +/- PRN | 65 [62,68] (n = 2) | 65 [62,68] (n = 2) |  |  |  |
|  | PRN only | 60 [32,80] (n = 9) | 60 [40,80] (n = 2) | 32 [24,36] (n = 3) | 80 [70,82] (n = 3) |  |
| Non-opioid NSAID | None | 38 [20,60] (n = 246) | 40 [20,60] (n = 104) | 20 [0,40] (n = 81) | 40 [20,60] (n = 44) | 55 [49,79] (n = 12) |
|  | Regular +/- PRN | 50 [38,70] (n = 31) | 50 [38,68] (n = 23) | 70 [70,80] (n = 3) | 30 [25,35] (n = 2) | 50 [38,60] (n = 3) |
|  | PRN only | 48 [28,69] (n = 58) | 40 [26,68] (n = 35) | 60 [40,70] (n = 17) | 35 [22,55] (n = 6) |  |
| Non-opioid paracetamol | None | 30 [10,60] (n = 175) | 40 [20,60] (n = 66) | 12 [0,40] (n = 62) | 30 [10,50] (n = 33) | 55 [51,71] (n = 10) |
|  | Regular +/- PRN | 50 [40,68] (n = 43) | 48 [30,56] (n = 32) | 60 [50,70] (n = 5) | 77 [68,78] (n = 3) | 70 [60,80] (n = 3) |
|  | PRN only | 40 [28,65] (n = 117) | 40 [25,61] (n = 64) | 50 [22,69] (n = 34) | 45 [40,60] (n = 16) | 12 [6,19] (n = 2) |
| Adjunctive | None | 34 [16,60] (n = 260) | 40 [20,60] (n = 113) | 25 [0,50] (n = 99) | 30 [20,50] (n = 39) | 55 [49,69] (n = 8) |
|  | Regular +/- PRN | 57 [40,75] (n = 58) | 50 [30,60] (n = 37) | 68 [64,71] (n = 2) | 74 [49,80] (n = 10) | 70 [55,75] (n = 5) |
|  | PRN only | 40 [25,50] (n = 17) | 40 [24,51] (n = 12) |  | 50 [40,60] (n = 3) | 25 [25,25] (n = 2) |

*VAS, visual analogue scale measured from 0-100mm.*

*Results are presented as* *median [IQR]. Index and all reviews are considered.*

*Participant with RDEB-PT is only included in the ‘Overall’ subtype category.*
